# Did previous involvement in research affect recruitment of young people with cerebral palsy to a longitudinal study of transitional health care?

**DOI:** 10.1136/bmjopen-2019-035525

**Published:** 2020-08-11

**Authors:** Elena Guiomar Garcia Jalón, Hanna Merrick, Allan Colver, Mark Linden, The Transition Collaborative Group, Caroline Bennett

**Affiliations:** 1 School of Social Sciences, Queen’s University Belfast, Belfast, UK; 2 Institute of Health and Society, Newcastle University, Newcastle upon Tyne, UK; 3 School of Nursing and Midwifery, Queen's University of Belfast, Belfast, UK

**Keywords:** statistics & research methods, paediatric neurology, community child health

## Abstract

**Objective:**

To assess whether being contacted about or participating in previous research and method of approaching potential participants affected recruitment to a transition study from child to adult healthcare services of young people with cerebral palsy (CP).

**Design and methods:**

Young people with CP aged 14–18 years without severe intellectual impairment were identified from regional registers of CP in Northern Ireland and the North of England. χ^2^ and Mann-Whitney U tests were used to assess differences in CP and sociodemographic characteristics between those recruited and those who refused. Logistic regression was used to assess contact about and recruitment to previous research and method of approach as predictors of recruitment, controlling for demographic and CP characteristics.

**Results:**

Of the 410 young people who were approached; 162 did not respond and of the 248 who responded, 96 (23%) were recruited. There were significant differences between those recruited and those who refused in age and number of previous studies they had participated in. Those who were older or who had previously been approached about research were more likely to be recruited to our study. However, those who had been recruited to previous studies were more likely to refuse to join our study.

**Conclusions:**

The method of approach to potential participants did not affect recruitment. Older adolescents and those who had been approached about previous research were more likely to take part in our study, although there was evidence of research fatigue because if they had actually been recruited to the previous studies they were less likely to join our study. Recruitment of adolescents to studies remains challenging.

**Strengths and limitations for this study**

- Study subjects were recruited from two population-based registers which used the same definition, severity and classification of cerebral palsy subtypes, and both confirmed information for cases at age 5 years.
- The study of the potential effect of prior involvement in research on recruitment was novel.
- Socioeconomic status could not be fully controlled for due to the lack of equivalence between the deprivation indices used in Northern Ireland and England.
- Analysis of the effect of previous involvement in research was limited to the number of studies; it could not take account of the type of study or the consent procedures.
- Young people who had been approached about or had taken part in three or more studies had to be treated as a single category due to small numbers.

## Introduction

There is little evidence about effective strategies for recruiting young people to research studies, in particular those with long-term conditions.^1–4^ Poorer health outcomes have been reported in this age group for non-responders to research invitations.^5^ One difficulty is that many adolescents may not want to be reminded of their condition or do not consider themselves to be ‘ill’.^6 7^ Problems with recruitment can lead to bias due to differences between those who consent and those who do not.^8–13^ Using disease-specific registers as sampling frames can reduce the risk of bias,^14^ but there may be issues with research fatigue if a register is used for many studies.

The Transition Research Programme (http://research.ncl.ac.uk/transition/index.html) includes a longitudinal study examining transition from child to adult healthcare services, with young people with cerebral palsy (CP) as one exemplar group. This longitudinal study offered the opportunity to evaluate potential differences in recruitment of young people with CP using two population-based UK registers for which participation in previous research was known, the North of England Collaborative CP Survey (NECCPS)^15^ and the Northern Ireland CP Register (NICPR).^16^ Equally, differences in the operating procedures of the two registers allowed assessment of the effect of Direct (through the researcher from the register) and Indirect (through a clinician known to the family) contacts with families on their decision to take part in research.

The goal of this study was to investigate whether being contacted about or participating in previous research and method of approaching potential participants (direct vs indirect) affected recruitment to a research study.

## Methods

### Patient and public involvement

Patients and the public were not involved in the design, conduct, reporting or dissemination of this particular study. However, the Transition Research Programme, which provided data for this study, included Representatives of patient organisations on the External Advisory Board and a young person working group which generated a publication.^17^

### Study design and participants

For the purpose of this study, we used the following definitions:

Transition study: the longitudinal study included in the Transition Research Programme which examined transition from child to adult healthcare services, with young people with CP as one exemplar group.

Potentially eligible: young people identified from two registers of CP, the NECCPS^15^ and the NICPR.^16^

Eligible: young people confirmed to meet the inclusion criteria.

Approached: young people approached about joining the transition study.

Not-approached: young people who could not be traced or the research team was advised not to approach.

Responders: those approached who responded; they either then declined to take part or were recruited into the transition study.

Non-responders: those approached who did not respond to the initial or follow-up letters.

Recruited: those approached who responded and consented to take part in the transition study.

Refusers: a combined group which included non-responders (passive refusal) and responders who declined to take part in the transition study (active refusal).

Direct approach: when young people and their families were contacted through the researcher from the register.

Indirect approach: when young people and their families were contacted through a clinician known to the family.

Young people were eligible for inclusion in the transition study if aged between 14 and 18 years 11 months, did not have severe intellectual impairment and could self-report. Young people were excluded if they were only seen in adult clinical services. Before approaching those potentially eligible, clinicians known to the family confirmed eligibility and contact details. All young people provided signed consent to join the transition study. For young people under 16 years of age, a parent also provided signed consent for their child to join the study.

Both the NECCPS and NICPR share a standardised definition and classification of CP; methods of ascertainment and data quality for each register have been described.^15 16^ Approach to the potentially eligible participants and their parents was undertaken differently for the two registers. The NECCPS is a consent-based register and therefore the researcher could contact potential participants directly by letter (direct approach). The NICPR follows an opt-out policy whereby children with CP are included unless families opt out. Thus, the NICPR asked clinicians known to the families to make the initial contact with subsequent contact by letter from the researcher (indirect approach). In both cases, two follow-up letters were sent if an initial response was not received.

### Data collected

Date of birth, sex, postcode, method and date of initial approach and decision about whether to join the transition study or not were recorded. Information was provided by the CP registers on the young people’s CP subtype, motor function using the gross motor function classification system (GMFCS)^18^ and intellectual ability as recorded when aged 4–5 years. The registers also provided information on the number of previous studies young people had been approached about and the number they had joined.

### Data analysis

Descriptive characteristics of the sample include frequencies, percentages, means and SD. Differences between recruited and refusers were assessed with χ2 test for categorical variables and Mann-Whitney U test for age. Differences between the subgroups within refusers, that is, non-responders (passive refusal) and responders who declined (active refusal), were also analysed. As passive and active refusal are different, it was deemed necessary to assess whether these two subgroups were different. Results from this analysis were used to define the dependent variable in the logistic regression analysis that was to follow.

Logistic regression using a standard or enter method assessed whether recruited versus refusers was predicted by method of approach (direct vs indirect) and/or the number of previous studies approached about or taken part in, controlling for: age when first approached, sex, home location and CP characteristics (subtype, GMFCS and intellectual impairment). Significance was set at p<0.05 and results are presented as ORs and 95% CIs. Goodness-of-fit was assessed using the Hosmer-Lemeshow test. Cox and Snell R Square and Nagelkerke R Square values were used as indicators of the variation in the dependent variable explained by the model. Analyses were performed in SPSS V.22.

## Results

**Figure 1:**
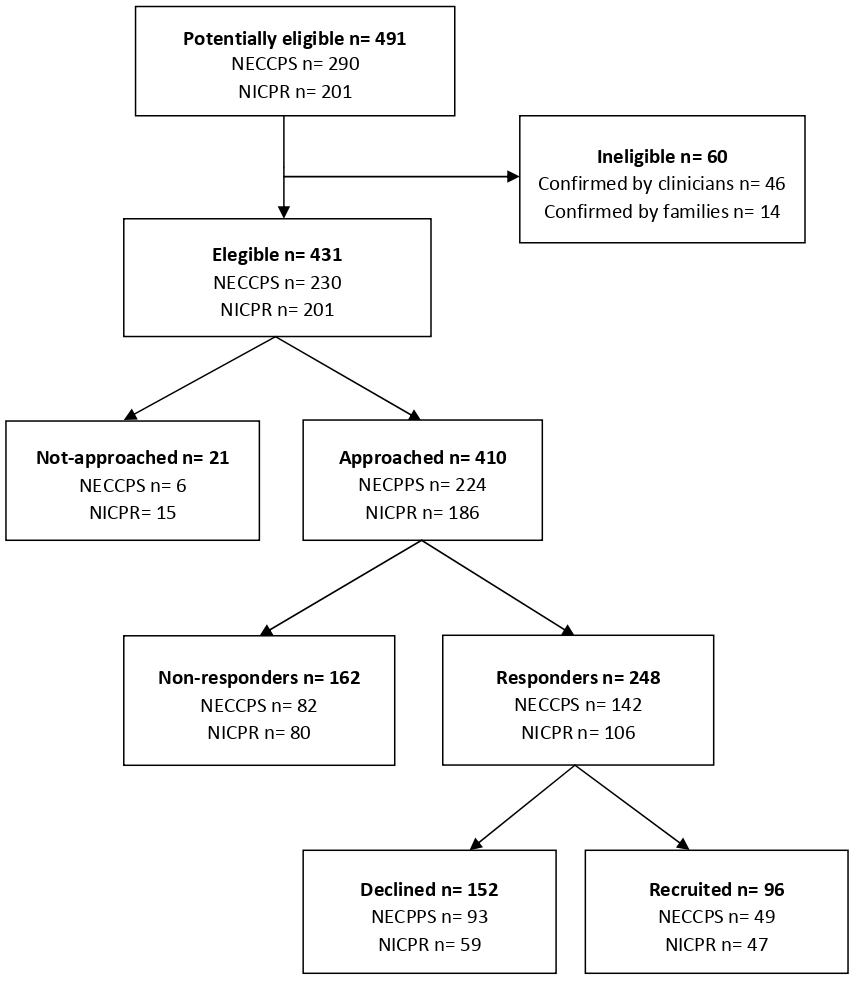
Recruitment flow chart. NECCPS, North of England Collaborative CP Survey;^15^ NICPR, Northern Ireland CP Register.

Figure 1 shows the recruitment flow to the transition study. A total of 491 young people with CP were identified from the NECCPS and NICPR. Of these, 60 were found to be ineligible by the family or their clinician because of severe intellectual impairment which had become manifest since age 5 years (n=57) or because the young person had already been transferred to adult services (n=3). Of the 431 eligible participants, 410 (95.1%) were approached. Of those who were not approached (n=21), 13 had moved away or could not be traced; three were not contacted following advice from clinicians known to the family and five due to other reasons. Of those approached, 162 (39.5%) did not respond. Of the remaining 248 (60.5%), 96 agreed to take part (23.4% of the 410 who were approached).

### Recruitment: recruited versus refusers; non-responders (passive refusal) versus responders who declined (active refusal)

Table 1 shows the breakdown of characteristics for those who were approached (n=410), those who were recruited (n=96) and refusers (n=314). Comparison between the two groups found a significant difference in age when approached (p=0.05), with the recruited being slightly younger (mean 16.1, SD=1.2) than refusers (mean 16.4, SD=1.4). There was also a significant difference in the number of previous studies they had taken part in (χ^2^=32.6, p<0.001), with 53.1% of recruited having taken part in one or more previous studies versus 32.2% of refusers. There were no significant differences for any of the other factors.

**Table 1:** Characteristics of the young people who were approached and comparison between recruited and refusers

Characteristic	Category	Approached n=410 (%)	Recruited n=96 (%)	Refusers n=314 (%)	χ^2^ (differences between recruited and refusers)
Sex	Male	245	53 (55.2%)	192 (61.1%)	χ^2^= 1.08 p=0.22
	Female	165	43 (44.8%)	122 (38.9%)	
Age when first approached	Mean (SD)	16.36 (1.34)	16.13 (1.22)	16.44 (1.37)	U†=17 032.5 p=0.05*
Home location	Urban	296	67 (69.8%)	229 (73.2%)	χ^2^= 0.42 p<0.51
	Rural	113	29 (30.2%)	84 (26.8%)	
	Missing	1			
CP subtype	Spastic unilateral	198 (48.3%)	45 (46.9%)	153 (48.7%)	χ^2^= 1.42 p=0.70
	Spastic bilateral	196 (47.8%)	48 (50.0%)	148 (47.1%)	
	Not spastic (dyskinesia, ataxia, unclassified)	16 (3.9%)	3 (3.1%)	13 (4.2%)	
GMFCS	Level I	106 (25.9%)	22 (23.4%)	84 (27.3%)	χ^2^= 1.31 p<0.86
	Level II	191 (46.6%)	44 (46.8%)	147 (47.7%)	
	Level III	65 (15.9%)	18 (19.1%)	47 (15.3%)	
	Level IV	26 (6.3%)	7 (7.4%)	19 (6.2%)	
	Level V	14 (3.4%)	3 (3.2%)	11 (3.6%)	
	Missing	8 (2.0%)			
Intellectual impairment	IQ>70	345 (84.1%)	81 (87.1%)	264 (85.4%)	χ^2^= 0.16 p<0.68
	IQ 50–69	57 (13.9%)	12 (12.9%)	45 (14.6%)	
	Missing	8 (2.0%)			
Approach method	Direct (NECCPS)	224	49 (51.0%)	175 (55.4%)	χ^2^= 0.65 p=0.41
	Indirect (NICPR)	186	47 (49.0%)	139 (44.3%)	
Approached about previous studies	0	153 (37.3%)	31 (32.3%)	122 (38.9%)	χ^2^= 10.19 p<0.11
	1	86 (21.0%)	/17 (17.7%)	69 (22.0%)	
	2	112 (27.3%)	31 (32.3%)	81 (25.8%)	
	>3	59 (14.4%)	17 (17.7%)	42 (13.3%)	
Recruited to previous studies	0	258 (62.9%)	45 (46.9%)	213 (67.8%)	χ^2^= 32.61 p<0.000*
	1	66 (16.1%)	16 (16.7%)	50 (15.9%)	
	2	56 (13.7%)	21 (21.9%)	35 (11.1%)	
	>3	30 (7.3%)	14 (14.5%)	16 (5.2%)	

*Significant difference.

†Mann-Whitney U test.

CP, cerebral palsy; GMFCS, gross motor function classification system; NECCPS, North of England Collaborative Cerebral Palsy Survey; NICPR, Northern Ireland Cerebral Palsy Register.

Analyses between the subgroups of non-responders (passive refusal, n=162) and responders who then declined (active refusal, n=152) showed no significant differences in any of the characteristics analysed.

### Predictors of recruitment to the transition study

The Hosmer-Lemeshow test found agreement between the observed and the predicted outcomes, indicating the full model was reliable (χ^2^=8.1; p=0.42). The full model was significant in distinguishing between refusers and recruited (χ^2^=36.8, p=0.004) and accounted for between 8.8% and 13.3% of the variance in refusal, with 96.7% of refusers successfully predicted. However, the model predicted only 8.6% of those recruited. Therefore, overall 76.2% of predictions were accurate. Table 2 shows ORs with 95% CIs for each predictor variable. Only age and the number of previous studies young people had been approached about or taken part in significantly predicted refusal. The OR values indicated that an increase of 1 year in age at contact was associated with a decrease in the odds of refusal by 0.79. OR values also indicated that being approached about many studies was associated with a decrease in the odds of refusal, especially if at least three studies (OR 0.11). In contrast, having been actually recruited to previous studies was associated with an increase in the odds of refusal, odds which rose with the number of studies recruited to.

**Table 2:** Factors associated with refusal to take part in the transition study: logistic regression

Variable	Category	OR (95% CI)
Method of approach	Indirect (NICPR)†	1.00
	Direct (NECCPS)	1.08 (0.5 to 2.32)
Sex	Female†	1.00
	Male	0.67 (0.4 to 1.12)
Age when first contacted		0.79 (0.63 to 0.98)*
Home location	Rural†	1.00
	Urban	0.83 (0.47 to 1.47)
CP subtype	Non-spastic†	1.00
	Spastic unilateral	1.6 (0.38 to 6.76)
	Spastic bilateral	1.55 (0.38 to 6.25)
GMFCS	Level V†	1.00
	Level I	0.67 (0.14 to 3.09)
	Level II	0.87 (0.19 to 3.8)
	Level III	1.09 (0.24 to 4.82)
	Level IV	0.96 (0.17 to 5.24)
Intellectual impairment	None†	1.00
	Yes	1.22 (0.56 to 2.67)
Approached about previous studies	No studies†	1.00
	One study	0.66 (0.29 to 1.5)
	Two studies	0.35 (0.11 to 1.14)
	Three or more studies	0.11 (0.02 to 0.69)*
Recruited to previous studies	No studies†	1.00
	One study	2.95 (1.1 to 7.91)*
	Two studies	7.45 (2.38 to 23.33)*
	Three or more studies	34.67 (6.345 to 189.45)*

*Significant predictor variables in the model p<0.05;.

†Reference category.

CP, cerebral palsy; GMFCS, gross motor function classification system; NECCPS, North of England Collaborative Cerebral Palsy Survey; NICPR, Northern Ireland Cerebral Palsy Register.

## Discussion

Of the 431 eligible young people, 96 (23.4%) were recruited to the transition study. The proportion of refusers was 76.6% (n=314) of whom 39.5% (n=162) did not respond and 37.1% (n=152) declined to take part. The majority who declined stated they were not interested in the study (n=83). Some young people had complex family or health situations and chose not to take part (n=6) and three young people declined because they had little or no contact with healthcare services.

Previous studies assessing recruitment bias and predictors for drop-out rate in surveys with children and young people with CP reported higher recruitment rates of between 72.6% and 47.3%.^14 19–21^ Although the refusal rate in those studies was lower, our non-response rate was similar to that reported by Dickinson *et al* (36.7%)^20^ and marginally higher than that reported by Parkes *et al* (31.5%).^19^

The Transition Research Programme involved young people as coresearchers^17^ who assisted with aspects of recruitment and retention of participants in the transition study, for example, the design of information sheets, consent and refusal forms and ensuring different routes of communication. Despite these efforts, more than half of those responders who declined reported lack of interest and more than one third of those approached did not respond. Although adult researchers and young people involved in the public and patient involvement (PPI) work for The Transition Research Programme described positive experiences,^17^ the impact of their work on recruitment to the transition study could not be assessed.

Transition from child to adult healthcare services has been described as a challenging time for young people with complex healthcare need resulting in issues with non-adherence to healthcare.^22 23^ This may not be different with research and some young people will not want to engage despite efforts to involve users in the recruitment process. There is evidence on the positive impact and challenges of PPI in health and social care research and its potential to increase recruitment of participants.^24 25^ However, there is a wide variation on how evidence was assessed and reported, and most studies only collected qualitative data.^24 25^ Nevertheless, researchers should consider various recruitment methods and ways in which users can assist with the recruitment of participants.^26 27^ It is also important for researchers to report all stages of recruitment^13^ and PPI.^4 24 25^ Compared with refusers, a higher percentage of those recruited to the transition study had taken part in one or more previous studies (53% vs 32%) and were younger on average. The only factors predicting refusal were age, the number of previous studies eligible participants had been approached about and the number of studies they had taken part in. Older individuals were less likely to refuse and this may be because they were more independent and making their own decisions. Also, the topic of the study may have been of more interest to those who were older. The information sharing and consent process used by researchers would have conveyed to the young people that their opinion was valued, thus increasing their sense of control.^6 28^ However, these findings should be considered with caution as data for age was non normally distributed, with a positive skewness towards younger age in the recruited group and negative skewness towards older age in the refusers group; this could explain the small difference in the mean age (0.3 years) between recruited and refusers which was only just significant (p=0.05). In other studies^14 19–21^ investigating recruitment of children and young people with CP, age was not a factor which affected recruitment, but inclusion criteria covered a wide age range (4–25 years) and included participants with all levels of intellectual ability. Indeed, authors reported that those with more complex or severe CP, such as the presence of intellectual problems or not being ambulant, were more likely to take part in their research.^21^ This may be because their parents gave consent, whereas in the transition study, the focus was on young people giving consent and young people with severe learning disabilities were excluded. This may have contributed to why the recruitment rate was lower than in previous studies. In addition, social networking and fitting in among peers has been shown to be important for adolescents; this may be relevant for those who may have refused to take part in a study simply because it reminds them of their condition.^6^

Research fatigue could also be an issue affecting recruitment. This study is the only one to our knowledge including information on the number of previous studies eligible participants had been approached about or taken part in. Our results showed that young people who had been previously approached about research were less likely to refuse to join our study, and this became even less likely as the number of studies they had been approached about rose. However, those actually recruited to previous research were more likely to refuse to join our study, with more studies making refusal even more likely. Approaching young people about studies can be positive as they may become more aware about research and thus more likely to want to participate in it. However, researchers should be aware of the potential for research fatigue among those recruited to previous studies. Registers such as the NECCPS and the NICPR would benefit from keeping records of studies they have been involved in, who has been contacted about research and their response.

There were no significant differences between recruited and refusers regarding the method of approach (direct vs indirect) and the logistic regression model did not find method of approach to be a factor predicting refusal. Therefore, it seems unlikely that different approach methods to contact eligible participants had influenced recruitment to our study. In contrast, previous research has shown that when parents and adolescents have discordant views, adolescents are more likely to agree to participate when approached by a clinician they know.^22^ Equally, Dickinson *et al* 20 found that registers which used the direct approach had half the refusal rate when compared with those using indirect methods. However, both NICPR and NECCPS are based in the UK, whereas Dickinson’s study involved registers from other countries (France, Italy, Sweden, Ireland and Denmark) with different data protection requirements.

The high non-response rate in this study (those who did not respond at all to the approach) is similar to rates reported in other studies.^14 19–21^ Thus, there may be a subset of young people with CP who are not interested in research. Further, there were no significant differences in any of their impairments or sociodemographics characteristics between those who declined (active refusal) and non-responders (passive refusal). Our model only predicted between 9% and 13.7% of the variance in recruitment, and therefore other health and socioeconomic factors may influence recruitment. Non-response has been linked to poorer health behaviour and differences in socioeconomic background but few studies have focused on adolescents.^4^ Mattila *et al* 5 29 found that non-responders to surveys among adolescents in Finland were more likely to engage in negative health behaviours, to suffer mental health disorders and to be more likely to die; these increased risks persisted to age 25 years. Although these studies focused on the general adolescent population, this developmental stage is also a challenging time in the lives of young people with chronic conditions regardless of their diagnoses.^30 31^ It is a time of particular risk for poorer medical outcomes and non-adherence to treatment.^32 33^ As previously discussed, young people may choose not to be involved in research. PPI has the potential to optimised recruitment, although evidence for this is limited.^24 25^

### Strengths and limitations

Both population-based registers used the same definition of CP, classification of CP subtypes and severity, and both confirmed information for cases at age 5 years.^13 14^ While this information was used initially to determine those who were potentially eligible, subsequent contact brought the information up to date and in particular we found that level of intellectual ability was often no longer accurate.

We could not fully assess how socioeconomic status affected recruitment because of the lack of equivalence between the deprivation indices used in Northern Ireland and England. However, it was possible to compare home location (urban vs rural). Young people who had been approached about or had taken part in three or more studies had to be analysed as a single category due to small numbers. Also, analysis of the effect of previous involvement in research was limited to the number of studies without taking into consideration their nature or the consent procedures. Although the p-value for those variables showing significant differences was well below p=0.05, there is always a possibility of type I errors.

## Conclusion

It is encouraging that the two different methods used to approach young people with CP (direct vs indirect) did not affect recruitment. Being older and approached about previous research were associated with increased likelihood of joining the transition study. However, even in this subset of young people of CP who were research aware and committed to being involved in studies, our findings showed evidence they could experience research fatigue. There is also a subset of young people with CP who are unwilling to take part in research as shown by the high non-response rate reported in this and previous studies. Recruitment of adolescents to research studies remains difficult.

## Supplementary Material

Reviewer comments

Author's manuscript
