# Supplementary material for: Did previous involvement in research affect recruitment of young people with cerebral palsy to a longitudinal study of transitional health care?
Source: BMJ Open. 2020 Aug 11;10(8):e035525. doi: 10.1136/bmjopen-2019-035525 (PMC7422630; doi:10.1136/bmjopen-2019-035525)
Supplement: Author's manuscript [file bmjopen-2019-035525.draft_revisions.pdf]

BMJ Open

BMJ Open is committed to open peer review. As part of this commitment we make the peer review history of every article we publish publicly available.

When an article is published we post the peer reviewers' comments and the authors' responses online. We also post the versions of the paper that were used during peer review. These are the versions that the peer review comments apply to.

The versions of the paper that follow are the versions that were submitted during the peer review process. They are not the versions of record or the final published versions. They should not be cited or distributed as the published version of this manuscript.

BMJ Open is an open access journal and the full, final, typeset and author-corrected version of record of the manuscript is available on our site with no access controls, subscription charges or pay-per-view fees (http://bmjopen.bmj.com).

If you have any questions on BMJ Open's open peer review process please email info.bmjopen@bmj.com

BMJ Open

Recruitment of young people with cerebral palsy in a longitudinal study of transitional health care

Journal:	*BMJ Open*
Manuscript ID	bmjopen-2019-035525
Article Type:	Original research
Date Submitted by the Author:	04-Nov-2019

Complete List of Authors:	Garcia Jalon, Elena Guiomar; School of Social Sciences, Queen's University, Belfast, UK,<br>Merrick, Hanna ; Newcastle University, Institute of Health and Society<br>Colver, Allan; Newcastle University, Institute of Health and Society<br>Linden, Mark; Queen's University of Belfast, School of Nursing and Midwifery

Keywords:	STATISTICS & RESEARCH METHODS, Paediatric neurology < NEUROLOGY, Community child health < PAEDIATRICS

SCHOLARONE™
Manuscripts

I, the Submitting Author has the right to grant and does grant on behalf of all authors of the Work (as defined in the below author licence), an exclusive licence and/or a non-exclusive licence for contributions from authors who are: i) UK Crown employees; ii) where BMJ has agreed a CC-BY licence shall apply, and/or iii) in accordance with the terms applicable for US Federal Government officers or employees acting as part of their official duties; on a worldwide, perpetual, irrevocable, royalty-free basis to BMJ Publishing Group Ltd ("BMJ") its licensees and where the relevant Journal is co-owned by BMJ to the co-owners of the Journal, to publish the Work in this journal and any other BMJ products and to exploit all rights, as set out in our licence.

The Submitting Author accepts and understands that any supply made under these terms is made by BMJ to the Submitting Author unless you are acting as an employee on behalf of your employer or a postgraduate student of an affiliated institution which is paying any applicable article publishing charge ("APC") for Open Access articles. Where the Submitting Author wishes to make the Work available on an Open Access basis (and intends to pay the relevant APC), the terms of reuse of such Open Access shall be governed by a Creative Commons licence – details of these licences and which Creative Commons licence will apply to this Work are set out in our licence referred to above.

Other than as permitted in any relevant BMJ Author's Self Archiving Policies, I confirm this Work has not been accepted for publication elsewhere, is not being considered for publication elsewhere and does not duplicate material already published. I confirm all authors consent to publication of this Work and authorise the granting of this licence.

For peer review only

TITLE: Recruitment of young people with cerebral palsy in a longitudinal study of transitional health care

AUTHORS: Elena Guiomar García Jalón, Hannah Merrick, Allan Colver, Mark Linden, on behalf of the Transition Collaborative Group[#]

CORRESPONDING AUTHOR:

Elena Guiomar García Jalón,
Affiliation: School of Social Sciences, Queen's University, Belfast, UK
Postal address: 9 Verdun Road, Eccles, Greater Manchester M30 8HE
Email: e.garciajalon@qub.ac.uk
Telephone number: 0044 (0)7849109149

CO-AUTHORS:

Hanna Merrick
Institute of Health and Society, Newcastle University, Newcastle upon Tyne, UK

Allan Colver
Institute of Health and Society, Newcastle University, Newcastle upon Tyne, UK

Mark Linden
School of Nursing and Midwifery, Queen's University of Belfast, Belfast, UK

WORD COUNT:
2,694

KEY WORDS:
Cerebral palsy, Adolescence, Recruitment, Transition

For peer review only

ABSTRACT

Objective: To assess whether being contacted about or participating in previous research affected recruitment to a Transition study from child to adult healthcare services of young people with cerebral palsy (CP).

Design and Methods: In this cross-sectional study young people with CP aged 14 to 18 years without severe intellectual impairment were identified from regional registers of CP in Northern Ireland and the North of England. Chi-square and Mann-Whitney U tests were used to assess differences in CP and socio-demographic characteristics between those recruited and those who refused. Logistic regression was used to assess predictors of recruitment, including demographic and CP characteristics, contact about and recruitment to previous research, method of approach.

Results: Of the 410 young people who were approached; 162 did not respond and of the 248 who responded, 96 (23%) were recruited. There were significant differences between those recruited and those who refused in age and number of previous studies they had participated in. Those who were older or who had previously been approached about research were more likely to be recruited to our study. However, those who had been recruited to previous studies were more likely to refuse to join our study.

Conclusions: The method of approach to potential participants did not affect recruitment. Older adolescents and those who had been approached about previous research were more likely to take part in our study, although there was evidence of research fatigue because if they had actually been recruited to the previous studies they were less likely to join our study. Recruitment of adolescents to studies remains challenging.

STRENGTHS AND LIMITATIONS

- Study subjects were recruited from two population-based registers which used the same definition, severity and classification of CP subtypes, and both confirmed information for cases at age 5 years.
- The study of the potential effect on recruitment of prior involved in research was novel.
- Socio-economic status could not be fully controlled for due to the lack of equivalence between the deprivation indices used in Northern Ireland and England
- Analysis of the effect of previous involvement in research was limited to the number of studies without taking into consideration their nature or the consent procedures.
- Young people who had been approached about or had taken part in three or more studies had to be analysed as a single category due to small numbers.

For peer review only - http://bmjopen.bmj.com/site/about/guidelines.xhtml

For peer review only

INTRODUCTION

There is little evidence about effective strategies for recruiting young people to research studies, in particular those with long term conditions.[1, 2, 3] Poorer health outcomes have been reported in this age group for non-responders to research invitations.[4] One difficulty is that many adolescents may not want to be reminded of their condition or do not consider themselves to be 'ill'.[5, 6] Problems with recruitment can lead to bias due to differences between those who consent and those who do not.[7-11] Using disease specific registers as sampling frames can reduce the risk of bias,[12] but there may be issues with research fatigue if a register is used for many studies.

The Transition Research Programme (http://research.ncl.ac.uk/transition/index.html) includes a longitudinal study examining transition from child to adult healthcare services, with young people with cerebral palsy (CP) as one exemplar group. This study offered the opportunity to evaluate potential differences in recruitment of young people with CP using two population-based UK registers for which participation in previous research was known, the North of England Collaborative CP Survey (NECCPS) [13] and the Northern Ireland CP Register (NICPR).[14] Differences in the operating procedures of the two registers also allowed assessment of the effect of Direct (through the researcher from the register) and Indirect (through a clinician known to the family) contact with families on their decision to take part in research.

We investigated whether being contacted about or participating in previous research affected recruitment to a research study.

METHODS

Ethics

The Transition study received a favourable ethics opinion from Newcastle and North Tyneside 1 Research Ethics Committee (12/NE/0059). All young people who agreed to take part in the Transition study provided written consent.

Patients and the public were not involved in the design, conduct, reporting or dissemination of this particular study. However the Transition Research Programme, which provided data for this study, included Representatives of patient organisations on the External Advisory Board and a young person working group which generated a publication.[15]

Study design and participants

For the purpose of this cross-sectional study we used the following definitions:

For peer review only - http://bmjopen.bmj.com/site/about/guidelines.xhtml

Potentially eligible: young people identified from two registers of CP, the NECCPS [13] and the NICPR.[14]

Eligible: young people confirmed to meet the inclusion criteria.

Approached: young people approached about joining the Transition study.

Not-approached: young people who could not be traced or the research team was advised not to approach.

Responders: those approached who responded; they either then declined to take part or were recruited into the Transition study.

Non-responders: those approached who did not respond to the initial or follow up letters.

Recruited: those approached who responded and consented to take part in the Transition study.

Refusers: a combined group which included Non-responders (passive refusal) and Responders who declined to take part in the Transition study (active refusal).

Direct approach: when young people and their families were contacted through the researcher from the register.

Indirect approach: when young people and their families were contacted through a clinician known to the family.

Young people were Eligible for inclusion in the Transition study if aged between 14 and 18 years 11 months, did not have severe intellectual impairment and could self-report. Young people were excluded if they were only seen in adult clinical services. Before approaching those Potentially eligible, clinicians known to the family confirmed eligibility and contact details.

Both the NECCPS and NICPR share a standardised definition and classification of CP; methods of ascertainment and data quality for each register have been described.[13, 14] Approach to the Potentially eligible participants and their parents was undertaken differently for the two registers. The NECCPS is a consent-based register and therefore the researcher could contact potential participants directly by letter (Direct approach). The NICPR follows an opt-out policy whereby children with CP are included unless families opt out. Thus, the NICPR asked clinicians known to the families to make the initial contact with subsequent contact by letter from the researcher (Indirect approach). In both cases, two follow-up letters were sent if an initial response was not received.

Data collected

Date of birth, sex, postcode, method and date of initial approach and decision about whether to join the Transition study or not were recorded. Information was provided by the CP registers on the young people's CP subtype, motor impairment using the Gross Motor Function Classification System (GMFCS) [16] and intellectual ability as recorded when aged 4-5 years. The registers also provided

For peer review only - http://bmjopen.bmj.com/site/about/guidelines.xhtml

information on the number of previous studies young people had been approached about and the number they had joined.

Data analysis

Descriptive characteristics of the sample include frequencies, percentages, means and standard deviations. Differences between Recruited and Refusers were assessed with chi-square test for categorical variables and Mann-Whitney U test for age. Differences between the sub-groups within Refusers, i.e. Non-responders (passive refusal) and Responders who declined (active refusal), were also analysed. Logistic regression assessed whether Recruited vs. Refusers was predicted by method of approach (Direct vs. Indirect) and/or the number of previous studies approached about or taken part in, controlling for: age when first approached, sex, home location and CP characteristics (subtype, GMFCS and intellectual impairment). Significance was set at $p < 0.05$and results are presented as odds ratios (ORs) and 95% confidence intervals (CIs). Goodness-of-fit was assessed using the Hosmer-Lemeshow test. Cox and Snell R Square and Nagelkerke R Square values were used as indicators of the variation in the dependent variable explained by the model. Analyses were performed in SPSS version 22.

RESULTS

Figure 1 shows the recruitment flow to the Transition study. A total of 491 young people with CP were identified from the NECCPS and NICPR. Of these, 60 were found to be ineligible by the family or their clinician because of severe intellectual impairment which had become manifest since age 5 years (n= 57) or because the young person had already been transferred to adult services (n= 3). Of the 431 eligible participants, 410 (95.1%) were approached. Of those who were not approached (n= 21) 13 had moved away or could not be traced; 3 were not contacted following advice from clinicians known to the family and 5 due to other reasons. Of those approached, 162 (39.5%) did not respond. Of the remaining 248 (60.5%), 96 agreed to take part (23.4% of the 410 who were approached).

Recruitment: Recruited vs. Refusers; Non-responders (passive refusal) vs. Responders who declined (active refusal)

Table 1 shows the breakdown of characteristics for those who were approached (n=410), those who were Recruited (n= 96) and Refusers (n= 314). Comparison between the two groups found a significant difference in age when approached (p= 0.05), with the Recruited being slightly younger (mean 16.1, SD= 1.2) than Refusers (mean 16.4, SD= 1.4). There was also a significant difference in the number of previous studies they had taken part in (χ^2= 32.6, p<0.001), with 53.1% of Recruited

For peer review only - http://bmjopen.bmj.com/site/about/guidelines.xhtml

having taken part in 1 or more previous studies vs.32.2% of Refusers. There were no significant differences for any of the other factors.

For peer review only

For peer review only - http://bmjopen.bmj.com/site/about/guidelines.xhtml

Table 1: Characteristics of the young people who were approached and comparison between Recruited and Refusers.

Characteristic	Category	Approached n= 410 (%)	Recruited n= 96 (%)	Refusers n= 314 (%)	χ^2 (differences between Recruited and Refusers)
**Sex**	Male	245	53 (55.2%)	192 (61.1%)	χ^2= 1.08 p= 0.22
	Female	165	43 (44.8%)	122 (38.9%)	
**Age when first approached**	Mean (SD)	16.36(1.34)	16.13 (1.22)	16.44 (1.37)	U[a]= 17,032.5 p= 0.05*
**Home location**	Urban	296	67 (69.8%)	229 (73.2%)	χ^2= 0.42 p< 0.51
	Rural	113	29 (30.2%)	84 (26.8%)	
	Missing	1			
**CP subtype**	Spastic unilateral	198 (48.3%)	45 (46.9%)	153 (48.7%)	χ^2= 1.42 p= 0.70
	Spastic bilateral	196 (47.8%)	48 (50.0%)	148 (47.1%)	
	Not spastic (dyskinesia, ataxia, unclassified)	16 (3.9%)	3 (3.1%)	13 (4.2%)	
**GMFCS**	Level I	106 (25.9%)	22 (23.4%)	84 (27.3%)	χ^2= 1.31 p< 0.86
	Level II	191 (46.6%)	44 (46.8%)	147 (47.7%)	
	Level III	65 (15.9%)	18 (19.1%)	47 (15.3%)	
	Level IV	26 (6.3%)	7 (7.4%)	19 (6.2%)	
	Level V	14 (3.4%)	3 (3.2%)	11 (3.6%)	
	Missing	8 (2.0%)			
**Intellectual impairment**	IQ >70	345 (84.1%)	81 (87.1%)	264 (85.4%)	χ^2= 0.16 p< 0.68
	IQ 50-69	57 (13.9%)	12 (12.9%)	45 (14.6%)	
	Missing	8 (2.0%)			
**Approach method**	Direct (NECCPS)	224	49 (51.0%)	175 (55.4%)	χ^2= 0.65 p= 0.41
	Indirect (NICPR)	186	47 (49.0%)	139 (44.3%)	
**Approached about previous studies**	0	153 (37.3%)	31 (32.3%)	122 (38.9%)	χ^2= 10.19 p< 0.11
	1	86 (21.0%)	17 (17.7%)	69 (22.0%)	
	2	112 (27.3%)	31 (32.3%)	81 (25.8%)	
	>3	59 (14.4%)	17 (17.7%)	42 (13.3%)	
**Recruited to previous studies**	0	258 (62.9%)	45 (46.9%)	213 (67.8%)	χ^2= 32.61 p< 0.000*
	1	66 (16.1%)	16 (16.7%)	50 (15.9%)	
	2	56 (13.7%)	21 (21.9%)	35 (11.1%)	
	>3	30 (7.3%)	14 (14.5%)	16 (5.2%)	

*Significant difference; [a]Mann-Whitney U test; NICPR Northern Ireland Cerebral Palsy Register; NECCPS North of England Cerebral Palsy Survey; GMFCS Gross Motor Function Classification System

For peer review only - http://bmjopen.bmj.com/site/about/guidelines.xhtml

Analyses between the sub-groups of Non-responders (passive refusal, n= 162) and Responders who then declined (active refusal, n= 152) showed no significant differences in any of the characteristics analysed.

Predictors of recruitment to the Transition study

The Hosmer-Lemeshow test found agreement between the observed and the predicted outcomes, indicating the full model was reliable (χ^2=8.1; p= 0.42). The full model was significant in distinguishing between Refusers and Recruited (χ^2=36.8, p=0.004) and accounted for between 8.8% and 13.3% of the variance in refusal, with 96.7% of Refusers successfully predicted. However, the model predicted only 8.6% of those Recruited. Therefore, overall 76.2% of predictions were accurate. Table 2 shows odds rations with 95% CIs for each predictor variable. Only age and the number of previous studies young people had been approached about or taken part in significantly predicted refusal. The ORs values indicated that an increase of 1 year in age at contact was associated with a decrease in the odds of refusal by 0.79. ORs values also indicated that being approached about many studies was associated with a decrease in the odds of refusal, especially if at least three studies (OR 0.11.) In contrast, having been actually recruited to previous studies was associated with an increase in the odds of refusal, odds which rose with the number of studies recruited to.

For peer review only - http://bmjopen.bmj.com/site/about/guidelines.xhtml

Table 2: Factors associated with refusal to take part in the Transition study: logistic regression

Variable	Category	Odds ratio (95% CI)
**Method of approach**	Indirect (NICPR)†	1.00
	Direct (NECCPS)	1.08 (0.5-2.32)
**Sex**	Female†	1.00
	Male	0.67 (0.4-1.12)
**Age when first contacted**		0.79 (0.63-0.98)*
**Home location**	Rural†	1.00
	Urban	0.83 (0.47-1.47)
**CP subtype**	Non-spastic†	1.00
	Spastic unilateral	1.6 (0.38-6.76)
	Spastic bilateral	1.55 (0.38-6.25)
**GMFCS**	Level V†	1.00
	Level I	0.67 (0.14-3.09)
	Level II	0.87 (0.19-3.8)
	Level III	1.09 (0.24-4.82)
	Level IV	0.96 (0.17-5.24)
**Intellectual impairment**	None†	1.00
	Yes	1.22 (0.56-2.67)
**Approached about previous studies**	No studies†	1.00
	One study	0.66 (0.29-1.5)
	Two studies	0.35 (0.11-1.14)
	Three or more studies	0.11 (0.02-0.69)*
**Recruited to previous studies**	No studies†	1.00
	One study	2.95 (1.1-7.91)*
	Two studies	7.45 (2.38-23.33)*
	Three or more studies	34.67 (6.345-189.45)*

*Significant predictor variables in the model $p < 0.05$; †Reference category; NICPR Northern Ireland Cerebral Palsy Register; NECCPS North of England Cerebral Palsy Survey; GMFCS Gross Motor Function Classification System

For peer review only - http://bmjopen.bmj.com/site/about/guidelines.xhtml

DISCUSSION

Of the 431 Eligible young people, 96 (23.4%) were recruited to the Transition study. The proportion of Refusers was 76.6% (n= 314) of whom 39.5% (n= 162) did not respond and 37.1% (n= 152) declined to take part. The majority who declined stated they were not interested in the study (n= 83). Some young people had complex family or health situations and chose not to take part (n= 6) and three young people declined because they had little or no contact with health care services. Previous studies assessing recruitment bias and predictors for drop-out rate in surveys with children and young people with CP reported higher recruitment rates of between 72.6% and 47.3%.[12, 17-19] Although the refusal rate in those studies was lower, our non-response rate was similar to that reported by Dickinson et al. (36.7%) [18] and marginally higher than that reported by Parkes et al. (31.5%). [17]

Compared to Refusers, a higher percentage of those Recruited to the Transition study had taken part in one or more previous studies and were younger on average (53% versus 32%). The only factors predicting refusal were age, the number of previous studies eligible participants had been approached about, and the number of studies they had taken part in. Older individuals were less likely to refuse and this may be because they were more independent and making their own decisions. Also, the topic of the study may have been of more interest to those who were older. The information sharing and consent process used by researchers would have conveyed to the young people that their opinion was valued, thus increasing their sense of control.[5, 20] However these findings should be considered with caution as the difference in age between those Recruited to the Transition study and those who Refused was only just significant (p= 0.05).

In other studies [12, 17-19] investigating recruitment of children and young people with CP, age was not a factor which affected recruitment, but inclusion criteria covered a wide age range (4-25 years) and included participants with all levels of intellectual ability. Indeed, authors reported that those with more complex or severe CP, such as the presence of intellectual problems or not being ambulant, were more likely to take part in their research.[19] This may be because their parents gave consent whereas in the Transition study the focus was on young people giving consent and young people with severe learning disabilities were excluded. This may have contributed to why the recruitment rate was lower than in previous studies. In addition, social networking and fitting in among peers has been shown to be important for adolescents; this may be relevant for those who may have refused to take part in a study simply because it reminds them of their condition.[5]

Research fatigue could also be an issue affecting recruitment. This study is the only one to our knowledge including information on the number of previous studies eligible participants had been

For peer review only - http://bmjopen.bmj.com/site/about/guidelines.xhtml

approached about or taken part in. Our results showed that young people who had been previously approached about research were less likely to refuse to join our study, and this became even less likely as the number of studies they had been approached about rose. However, those actually recruited to previous research were more likely to refuse to join our study, with more studies making refusal even more likely. Approaching young people about studies can be positive as they may become more aware about research and thus more likely to want to participate in it. However, researchers should be aware of the potential for research fatigue among those recruited to previous studies. Registers such as the NECCPS and the NICPR would benefit from keeping records of studies they have been involved in, who has been contacted about research and their response.

There were no significant differences between Recruited and Refusers regarding the method of approach (Direct vs. Indirect) and the logistic regression model did not find method of approach to be a factor predicting refusal. Therefore, it seems unlikely that different approach methods to contact eligible participants had influenced recruitment to our study. In contrast, previous research has shown that when parents and adolescents have discordant views, adolescents are more likely to agree to participate when approached by a clinician they know.[20] Equally, Dickinson et al. [18] found that registers which used the Direct approach had half the refusal rate when compared to those using Indirect methods. However, both NICPR and NECCPS are based in the UK whereas Dickinson's study involved registers from other countries (France, Italy, Sweden, Republic of Ireland and Denmark) with different data protection requirements.

The high non-response rate in this study (those who did not respond at all to the approach) is, similar to rates reported in other studies.[12, 17-19] Thus, there may be a subset of young people with CP who are not interested in research. Further, there were no significant differences in any of their impairments or socio-demographics characteristics between those who declined (active refusal) and Non-responders (passive refusal). Our model only predicted between 9% and 13.7% of the variance in recruitment, and therefore other health and socio-economic factors may influence recruitment. Non-response has been linked to poorer health behaviour and differences in socio-economic background but few studies have focused on adolescents.[4] Mattila et al. [4,21] found that non-responders to surveys among adolescents in Finland were more likely to engage in negative health behaviours, to suffer mental health disorders and to be more likely to die; these increased risks persisted to age 25 years. Although these studies focused on the general adolescent population, this developmental stage is also a challenging time in the lives of young people with chronic conditions regardless of their diagnoses.[22, 23] It is a time of particular risk for poorer medical outcomes and non-adherence to treatment.[24, 25]

For peer review only - http://bmjopen.bmj.com/site/about/guidelines.xhtml

Strengths and limitations

Both population-based registers used the same definition of CP, classification of CP subtypes and severity, and both confirmed information for cases at age 5 years.[13, 14] Whilst this information was used initially to determine those who were Potentially eligible, subsequent contact brought the information up to date and in particular we found that level of intellectual ability was often no longer accurate.

We could not fully assess how socio-economic status affected recruitment because of the lack of equivalence between the deprivation indices used in Northern Ireland and England. However, it was possible to compare home location (urban vs. rural). Young people who had been approached about or had taken part in three or more studies had to be analysed as a single category due to small numbers. Also, analysis of the effect of previous involvement in research was limited to the number of studies without taking into consideration their nature or the consent procedures. Although the p value for those variables showing significant differences was well below p=0.05, there is always a possibility of type I errors.

CONCLUSION

It is encouraging that the two different methods used to approach young people with CP (Direct vs. Indirect) did not affect recruitment. Being older and approached about previous research were associated with increased likelihood of joining the Transition study. However, even in this subset of young people of CP who were research aware and committed to being involved in studies, our findings showed evidence they could experience research fatigue. There is also a subset of young people with CP who are unwilling to take part in research as shown by the high non-response rate reported in this and previous studies. Recruitment of adolescents to research studies remains difficult.

WHAT THIS PAPER ADDS

- Increased age and previous contact about or participation in research were statistically significant predictors ($p < 0.05$) of recruitment to the Transition study.
- Whilst previous involvement of young people in research seems to raise their interest in subsequent studies, research fatigue may prevent translation of their interest into actual participation in a new study.
- Different method of approach to contact eligible participants used in the Transition study (Direct vs. Indirect) did not influenced recruitment.

For peer review only - http://bmjopen.bmj.com/site/about/guidelines.xhtml

ACKNOWLEDGEMENTS

#The Transition Collaborative Group consists of: the authors of this paper; other co-applicants: Caroline Bennett, Gail Dovey-Pearce, Ann Le Couteur, Helen McConachie, Janet McDonagh, Jeremy Parr, Mark Pearce, Tim Rapley, Debbie Reape, Luke Vale; advisors: Nichola Chater, Helena Gleeson; local investigators: Anastasia Bem, Stuart Bennett, Amanda Billson, Stephen Bruce, Tim Cheetham, Diana Howlett, Zilla Huma, Maria Lohan, Melanie Meek, Jenny Milne, Julie Owens, NanduThalange.

We acknowledge the support of the National Institute for Health Research Clinical Research Network. We are grateful to the young people and their families who participated in the study. We thank the research assistants for their enthusiasm and dedication to contacting young people and collecting high-quality data: Kamar Ameen-Ali, Charlotte George, Kate Hardenberg, Holly Roper, Tracy Scott, Louise Ting, Rose Watson, Hazel Windmill; and we thank Richard Hardy, Alison Mulvenna and Sarah Nolan for software support and administrative support.

FUNDING

This paper summarises independent research funded by the National Institute for Health Research (NIHR) under its Programme Grants for Applied Research scheme (RP-PG-0610-10112). The views expressed are those of the authors and not necessarily those of the UK National Health Service, the NIHR or the Department of Health.

CONFLICT OF INTEREST

Potential Conflicts of Interest: The authors have no conflicts of interest relevant to this article to disclose

AUTHOR STATEMENT

Authors' contributions: Elena Guiomar García Jalón, Hannah Merrick, Allan Colver, Mark Linden participated in the planning of the study. Elena Guiomar García Jalón had responsibility for data collection with the collaboration of Hannah Merrick. Elena Guiomar García Jalón was responsible for planning and conducting data analysis with the collaboration of Hannah Merrick and Allan Colver. Elena Guiomar García Jalón wrote the drafts of the manuscript with input by Hannah Merrick, Allan Colver, Mark Linden. All authors have seen and approved the version to be published.

REFERENCES

1. Spigarelli M. Adolescent participation in research. *J Adolesc Health* 2008; 43: 1-2.

2. Middelkoop K, Landon M, Mark D, et al. Adolescent and Adult Participation in an HIV vaccine trial preparedness cohort in South Africa. *J Adolesc Health* 2008; 43: 8-14.

For peer review only - http://bmjopen.bmj.com/site/about/guidelines.xhtml

3. Hendricks-Fergunson VL, Cherve BO, Burns DS, et al. Recruitment strategies and rates of a multi-site behavioural intervention for adolescents and young adults with cancer. *J Pediatr Health Care* 2012; 27: 434-42.

4. Mattila VM, Parkkari J, Rimpela A. Adolescent survey non-response and later risk of death. A prospective cohort study of 78,609 persons with 11-year follow-up. *BMC Public Health* 2007; 7: 87.

5. McDonagh JE, Bateman B. Nothing about us without us: considerations for research involving young people. *Arch Dis Child (Education and Practice)* 2012; 97: 55-60.

6. van Staa A, Jedeloo S, Latour JM, et al. Exciting but exhausting: experiences with participatory research with chronically ill adolescents. *Health Expec* 2010; 13(1):95-107.

7. McCullagh S, Feinstein A. Outcome after mild traumatic brain injury: an examination of recruitment bias. *J NeurolNeurosur Psych* 2003; 74: 39-43.

8. Rahi JS, Manara I, Tuomainen H, et al. Engaging families in health services research on childhood visual impairment: barriers to, and degree and nature of bias in, participation. *Brit J Ophthalmol* 2004; 88: 782-7.

9. Al-Shahi R, Vousden C, Warlow C, for the Scottish Intracranial Vascular Malformation Study (SIVMS) Steering Committee. Bias from requiring explicit consent from all participants in observational research: prospective, population based study. *BMJ* 2005; 31: 942-5.

10. Hille ETM, Elbertse L, Gravenhorst JB, et al. Non-response bias in a follow-up study of 19-year-old adolescents born as preterm infants. *Pediatrics* 2005; 116: 622-66.

11. Junghans C, Feder G, Hemingway H, et al. Recruiting patients to medical research: double blind randomized trial of 'opt-in' versus 'opt-out' strategies. *BMJ* 2005; 33: 940-2.

12. Parkes J, Kerr C, McDowell BC, et al. Recruitment bias in a population-based study of children with cerebral palsy. *Pediatrics* 2006; 118: 1616-22.

13. Colver AF, Gibson M, Hey EN, et al. Increasing rates of cerebral palsy across the severity spectrum in north-east England 1964-1993. The North of England Collaborative Cerebral Palsy Survey. *Arch Dis Child*. *Fetal Neonatal Edition* 2000; 83:F7–12.

14. Parkes J, Dolk H, Hill N, et al. Cerebral palsy in Northern Ireland: 1981-93. *Paediatr Perinat Ep* 2001; 15: 278-86.

For peer review only - http://bmjopen.bmj.com/site/about/guidelines.xhtml

15. Dovey-Pearce G, Walker S, Fairgrieve S, et al. The burden of proof: The process of involving young people in research. *Health Expect* 2019; 00: 1-10.

16. Palisano R, Rosenbaum P, Walter S, et al. Development and reliability of a system to classify gross motor function in children with cerebral palsy. *Dev Med Child Neurol* 1997; 39: 214–23.

17. Parkes J, Donnelly C, McDowell B, et al. Recruitment bias and characteristics of participants with severe cerebral palsy in a cross-sectional survey. *J AdvNurs* 2012; 68: 368–78.

18. Dickinson H, Parkinson K, McManus V, et al. Assessment of data quality in a multi-centre cross-sectional study of participation and quality of life of children with cerebral palsy. *BMC Public Health* 2006; 6: 273.

19. Dickinson HO, Rapp M, Arnaud C, et al. Predictors of drop-out in a multi-centre longitudinal study of participation and quality of life of children with cerebral palsy. *BMC Research Notes* 2012*;* 15: 300.

20. Brody JL, Annett RD, Scherer DG, et al. Enrolling adolescents in asthma research: adolescent, parent and physician influence in the decision-making process. *J Asthma* 2009; 49: 492-7.

21. Mattila V, Parkkari J, Kannus P, et al. Occurrence and risk factors of unintentional injuries among 12 to 18 year old Finns – a survey of 8219 adolescents. *Eur J Epidemiol* 2004; 19: 437-44.

22. Bolton-Maggs PH. Transition of care from paediatric to adult services in haematology. *Arch Dis Child* 2007; 92: 797-801.

23. JordanA, McDonagh JE. Recognition of emerging adulthood in UK rheumatology: the case for young adult rheumatology service developments. *Rheumatology (Oxford)* 2007; 46: 188-91.

24. Reed-Knight B, Blount RL, Gilleland J. The transition of health care responsibility from parents to youth diagnosed with chronic illness: adevelopmentalsystemsperspective. *Fam Syst Health* 2014; 32: 219-34.

25. Fredericks EM, Magee JC, Eder SJ, et al. Quality improvement targeting adherence during the transition from a pediatric to adult liver transplant clinic. *J ClinPsychol Med Settings* 2015; 22: 150-9.

For peer review only - http://bmjopen.bmj.com/site/about/guidelines.xhtml

Figure 1 Recruitment flow chart

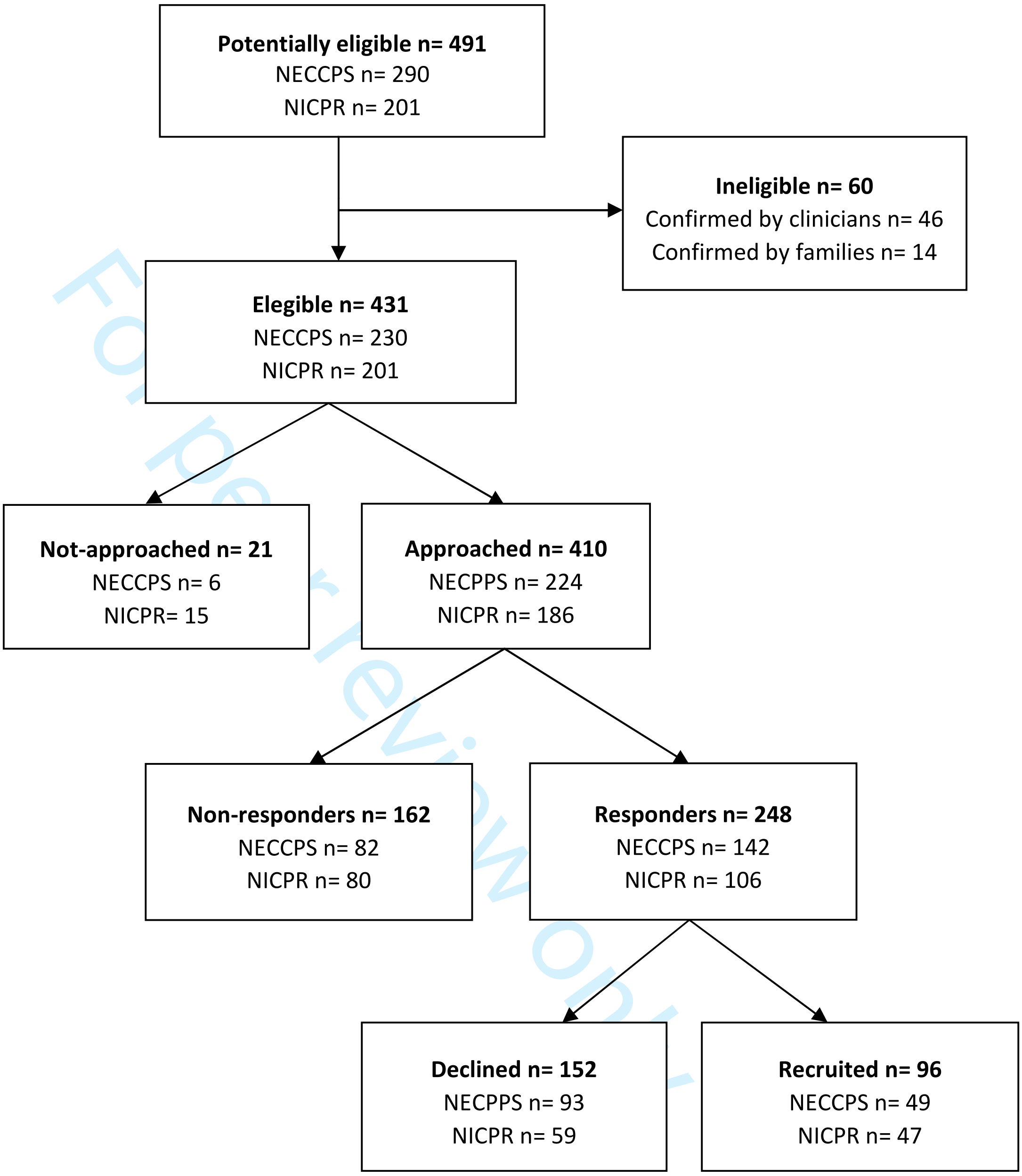

NICPR Northern Ireland Cerebral Palsy Register – Indirect approach
NECCPS North of England Cerebral Palsy Survey – Direct approach

STROBE Statement—Checklist of items that should be included in reports of ***cross-sectional studies***

	Item No	Recommendation	Page No
**Title and abstract**	1	(*a*) Indicate the study's design with a commonly used term in the title or the abstract	1
		(*b*) Provide in the abstract an informative and balanced summary of what was done and what was found	2
**Introduction**			
Background/rationale	2	Explain the scientific background and rationale for the investigation being reported	3
Objectives	3	State specific objectives, including any prespecified hypotheses	3
**Methods**			
Study design	4	Present key elements of study design early in the paper	3
Setting	5	Describe the setting, locations, and relevant dates, including periods of recruitment, exposure, follow-up, and data collection	3-4
Participants	6	(*a*) Give the eligibility criteria, and the sources and methods of selection of participants	3-4
Variables	7	Clearly define all outcomes, exposures, predictors, potential confounders, and effect modifiers. Give diagnostic criteria, if applicable	4
Data sources/ measurement	8*	For each variable of interest, give sources of data and details of methods of assessment (measurement). Describe comparability of assessment methods if there is more than one group	4
Bias	9	Describe any efforts to address potential sources of bias	-
Study size	10	Explain how the study size was arrived at	5
Quantitative variables	11	Explain how quantitative variables were handled in the analyses. If applicable, describe which groupings were chosen and why	4
Statistical methods	12	(*a*) Describe all statistical methods, including those used to control for confounding	5
		(*b*) Describe any methods used to examine subgroups and interactions	5
		(*c*) Explain how missing data were addressed	-
		(*d*) If applicable, describe analytical methods taking account of sampling strategy	NA
		(*e*) Describe any sensitivity analyses	-
**Results**			
Participants	13*	(a) Report numbers of individuals at each stage of study—eg numbers potentially eligible, examined for eligibility, confirmed eligible, included in the study, completing follow-up, and analysed	5
		(b) Give reasons for non-participation at each stage	5
		(c) Consider use of a flow diagram	Separate file uploaded
Descriptive data	14*	(a) Give characteristics of study participants (eg demographic, clinical, social) and information on exposures and potential confounders	6
		(b) Indicate number of participants with missing data for each variable of interest	6
Outcome data	15*	Report numbers of outcome events or summary measures	6

For peer review only

Main results	16	(*a*) Give unadjusted estimates and, if applicable, confounder-adjusted estimates and their precision (eg, 95% confidence interval). Make clear which confounders were adjusted for and why they were included	5, 7, 8
		(*b*) Report category boundaries when continuous variables were categorized	
		(*c*) If relevant, consider translating estimates of relative risk into absolute risk for a meaningful time period	
Other analyses	17	Report other analyses done—eg analyses of subgroups and interactions, and sensitivity analyses	7
**Discussion**			
Key results	18	Summarise key results with reference to study objectives	9-10
Limitations	19	Discuss limitations of the study, taking into account sources of potential bias or imprecision. Discuss both direction and magnitude of any potential bias	11
Interpretation	20	Give a cautious overall interpretation of results considering objectives, limitations, multiplicity of analyses, results from similar studies, and other relevant evidence	11
Generalisability	21	Discuss the generalisability (external validity) of the study results	-
**Other information**			
Funding	22	Give the source of funding and the role of the funders for the present study and, if applicable, for the original study on which the present article is based	12

*Give information separately for exposed and unexposed groups.

Note: An Explanation and Elaboration article discusses each checklist item and gives methodological background and published examples of transparent reporting. The STROBE checklist is best used in conjunction with this article (freely available on the Web sites of PLoS Medicine at http://www.plosmedicine.org/, Annals of Internal Medicine at http://www.annals.org/, and Epidemiology at http://www.epidem.com/). Information on the STROBE Initiative is available at www.strobe-statement.org.

For peer review only

BMJ Open

Did previous involvement in research affect recruitment of young people with cerebral palsy to a longitudinal study of transitional health care?

Journal:	*BMJ Open*
Manuscript ID	bmjopen-2019-035525.R1
Article Type:	Original research
Date Submitted by the Author:	22-Apr-2020
Complete List of Authors:	Garcia Jalon, Elena Guiomar; School of Social Sciences, Queen’s University, Belfast, UK,<br>Merrick, Hanna ; Newcastle University, Institute of Health and Society<br>Colver, Allan; Newcastle University, Institute of Health and Society<br>Linden, Mark; Queen's University of Belfast, School of Nursing and Midwifery
**Primary Subject Heading**:	Research methods
Secondary Subject Heading:	Health services research, Paediatrics
Keywords:	STATISTICS & RESEARCH METHODS, Paediatric neurology < NEUROLOGY, Community child health < PAEDIATRICS

SCHOLARONE™
Manuscripts

I, the Submitting Author has the right to grant and does grant on behalf of all authors of the Work (as defined in the below author licence), an exclusive licence and/or a non-exclusive licence for contributions from authors who are: i) UK Crown employees; ii) where BMJ has agreed a CC-BY licence shall apply, and/or iii) in accordance with the terms applicable for US Federal Government officers or employees acting as part of their official duties; on a worldwide, perpetual, irrevocable, royalty-free basis to BMJ Publishing Group Ltd ("BMJ") its licensees and where the relevant Journal is co-owned by BMJ to the co-owners of the Journal, to publish the Work in this journal and any other BMJ products and to exploit all rights, as set out in our licence.

The Submitting Author accepts and understands that any supply made under these terms is made by BMJ to the Submitting Author unless you are acting as an employee on behalf of your employer or a postgraduate student of an affiliated institution which is paying any applicable article publishing charge ("APC") for Open Access articles. Where the Submitting Author wishes to make the Work available on an Open Access basis (and intends to pay the relevant APC), the terms of reuse of such Open Access shall be governed by a Creative Commons licence – details of these licences and which Creative Commons licence will apply to this Work are set out in our licence referred to above.

Other than as permitted in any relevant BMJ Author's Self Archiving Policies, I confirm this Work has not been accepted for publication elsewhere, is not being considered for publication elsewhere and does not duplicate material already published. I confirm all authors consent to publication of this Work and authorise the granting of this licence.

For peer review only

TITLE: Did previous involvement in research affect recruitment of young people with cerebral palsy to a longitudinal study of transitional health care?

AUTHORS: Elena Guiomar García Jalón, Hannah Merrick, Allan Colver, Mark Linden, on behalf of the Transition Collaborative Group#

CORRESPONDING AUTHOR:

Elena Guiomar García Jalón,
Affiliation: School of Social Sciences, Queen's University, Belfast, UK
Postal address: 9 Verdun Road, Eccles, Greater Manchester M30 8HE
Email: e.garciajalon@qub.ac.uk
Telephone number: 0044 (0)7849109149

CO-AUTHORS:

Hannah Merrick
Institute of Health and Society, Newcastle University, Newcastle upon Tyne, UK

Allan Colver
Institute of Health and Society, Newcastle University, Newcastle upon Tyne, UK

Mark Linden
School of Nursing and Midwifery, Queen's University of Belfast, Belfast, UK

WORD COUNT:
3,029

KEY WORDS:
Cerebral palsy, Adolescence, Recruitment, Transition

For peer review only

For peer review only - http://bmjopen.bmj.com/site/about/guidelines.xhtml

ABSTRACT

Objective: To assess whether being contacted about or participating in previous research and method of approaching potential participants affected recruitment to a Transition study from child to adult healthcare services of young people with cerebral palsy (CP).

Design and Methods: Young people with CP aged 14 to 18 years without severe intellectual impairment were identified from regional registers of CP in Northern Ireland and the North of England. Chi-square and Mann-Whitney U tests were used to assess differences in CP and socio-demographic characteristics between those recruited and those who refused. Logistic regression was used to assess contact about and recruitment to previous research and method of approach as predictors of recruitment, controlling for demographic and CP characteristics.

Results: Of the 410 young people who were approached; 162 did not respond and of the 248 who responded, 96 (23%) were recruited. There were significant differences between those recruited and those who refused in age and number of previous studies they had participated in. Those who were older or who had previously been approached about research were more likely to be recruited to our study. However, those who had been recruited to previous studies were more likely to refuse to join our study.

Conclusions: The method of approach to potential participants did not affect recruitment. Older adolescents and those who had been approached about previous research were more likely to take part in our study, although there was evidence of research fatigue because if they had actually been recruited to the previous studies they were less likely to join our study. Recruitment of adolescents to studies remains challenging.

STRENGTHS AND LIMITATIONS

- Study subjects were recruited from two population-based registers which used the same definition, severity and classification of CP subtypes, and both confirmed information for cases at age 5 years.
- The study of the potential effect of prior involvement in research on recruitment was novel.
- Socio-economic status could not be fully controlled for due to the lack of equivalence between the deprivation indices used in Northern Ireland and England
- Analysis of the effect of previous involvement in research was limited to the number of studies; it could not take account of the type of study or the consent procedures.
- Young people who had been approached about or had taken part in three or more studies had to be treated as a single category due to small numbers.

For peer review only - http://bmjopen.bmj.com/site/about/guidelines.xhtml

INTRODUCTION

There is little evidence about effective strategies for recruiting young people to research studies, in particular those with long term conditions.[1, 2, 3, 4] Poorer health outcomes have been reported in this age group for non-responders to research invitations.[5] One difficulty is that many adolescents may not want to be reminded of their condition or do not consider themselves to be 'ill'.[6, 7] Problems with recruitment can lead to bias due to differences between those who consent and those who do not.[8-13] Using disease specific registers as sampling frames can reduce the risk of bias,[14] but there may be issues with research fatigue if a register is used for many studies.

The Transition Research Programme (http://research.ncl.ac.uk/transition/index.html) includes a longitudinal study examining transition from child to adult healthcare services, with young people with cerebral palsy (CP) as one exemplar group. This longitudinal study offered the opportunity to evaluate potential differences in recruitment of young people with CP using two population-based UK registers for which participation in previous research was known, the North of England Collaborative CP Survey (NECCPS) [15] and the Northern Ireland CP Register (NICPR).[16] Equally, differences in the operating procedures of the two registers allowed assessment of the effect of Direct (through the researcher from the register) and Indirect (through a clinician known to the family) contact with families on their decision to take part in research.

The goal of this study was to investigate whether being contacted about or participating in previous research and method of approaching potential participants (direct vs. indirect) affected recruitment to a research study.

METHODS

Ethics

The Transition study received a favourable ethics opinion from Newcastle and North Tyneside 1 Research Ethics Committee (12/NE/0059). All young people who agreed to take part in the Transition study provided written consent.

Patient and public involvement

Patients and the public were not involved in the design, conduct, reporting or dissemination of this particular study. However, the Transition Research Programme, which provided data for this study, included Representatives of patient organisations on the External Advisory Board and a young person working group which generated a publication.[17]

For peer review only - http://bmjopen.bmj.com/site/about/guidelines.xhtml

Study design and participants

For the purpose of this study we used the following definitions:

Transition study: the longitudinal study included in the Transition Research Programme which examined transition from child to adult healthcare services, with young people with CP as one exemplar group

Potentially eligible: young people identified from two registers of CP, the NECCPS [15] and the NICPR.[16]

Eligible: young people confirmed to meet the inclusion criteria.

Approached: young people approached about joining the Transition study.

Not-approached: young people who could not be traced or the research team was advised not to approach.

Responders: those approached who responded; they either then declined to take part or were recruited into the Transition study.

Non-responders: those approached who did not respond to the initial or follow up letters.

Recruited: those approached who responded and consented to take part in the Transition study.

Refusers: a combined group which included Non-responders (passive refusal) and Responders who declined to take part in the Transition study (active refusal).

Direct approach: when young people and their families were contacted through the researcher from the register.

Indirect approach: when young people and their families were contacted through a clinician known to the family.

Young people were Eligible for inclusion in the Transition study if aged between 14 and 18 years 11 months, did not have severe intellectual impairment and could self-report. Young people were excluded if they were only seen in adult clinical services. Before approaching those Potentially eligible, clinicians known to the family confirmed eligibility and contact details. All young people provided signed consent to join the Transition study. For young people under sixteen years of age, a parent also provided signed consent for their child to join the study.

Both the NECCPS and NICPR share a standardised definition and classification of CP; methods of ascertainment and data quality for each register have been described.[15, 16] Approach to the Potentially eligible participants and their parents was undertaken differently for the two registers. The NECCPS is a consent-based register and therefore the researcher could contact potential participants directly by letter (Direct approach). The NICPR follows an opt-out policy whereby children with CP are included unless families opt out. Thus, the NICPR asked clinicians known to the

For peer review only - http://bmjopen.bmj.com/site/about/guidelines.xhtml

families to make the initial contact with subsequent contact by letter from the researcher (Indirect approach). In both cases, two follow-up letters were sent if an initial response was not received.

Data collected

Date of birth, sex, postcode, method and date of initial approach and decision about whether to join the Transition study or not were recorded. Information was provided by the CP registers on the young people's CP subtype, motor function using the Gross Motor Function Classification System (GMFCS) [18] and intellectual ability as recorded when aged 4-5 years. The registers also provided information on the number of previous studies young people had been approached about and the number they had joined.

Data analysis

Descriptive characteristics of the sample include frequencies, percentages, means and standard deviations. Differences between Recruited and Refusers were assessed with chi-square test for categorical variables and Mann-Whitney U test for age. Differences between the sub-groups within Refusers, i.e. Non-responders (passive refusal) and Responders who declined (active refusal), were also analysed. As passive and active refusal are different, it was deemed necessary to assess whether these two subgroups were different. Results from this analysis were used to define the dependent variable in the logistic regression analysis that was to follow.

Logistic regression using a standard or enter method assessed whether Recruited vs. Refusers was predicted by method of approach (Direct vs. Indirect) and/or the number of previous studies approached about or taken part in, controlling for: age when first approached, sex, home location and CP characteristics (subtype, GMFCS and intellectual impairment). Significance was set at $p < 0.05$and results are presented as odds ratios (ORs) and 95% confidence intervals (CIs). Goodness-of-fit was assessed using the Hosmer-Lemeshow test. Cox and Snell R Square and Nagelkerke R Square values were used as indicators of the variation in the dependent variable explained by the model. Analyses were performed in SPSS version 22.

RESULTS

Figure 1 shows the recruitment flow to the Transition study. A total of 491 young people with CP were identified from the NECCPS and NICPR. Of these, 60 were found to be ineligible by the family or their clinician because of severe intellectual impairment which had become manifest since age 5 years (n= 57) or because the young person had already been transferred to adult services (n= 3). Of the 431 eligible participants, 410 (95.1%) were approached. Of those who were not approached (n= 21) 13 had moved away or could not be traced; 3 were not contacted following advice from

For peer review only - http://bmjopen.bmj.com/site/about/guidelines.xhtml

clinicians known to the family and 5 due to other reasons. Of those approached, 162 (39.5%) did not respond. Of the remaining 248 (60.5%), 96 agreed to take part (23.4% of the 410 who were approached).

Recruitment: Recruited vs. Refusers; Non-responders (passive refusal) vs. Responders who declined (active refusal)

Table 1 shows the breakdown of characteristics for those who were approached (n=410), those who were Recruited (n= 96) and Refusers (n= 314). Comparison between the two groups found a significant difference in age when approached (p= 0.05), with the Recruited being slightly younger (mean 16.1, SD= 1.2) than Refusers (mean 16.4, SD= 1.4). There was also a significant difference in the number of previous studies they had taken part in (χ^2= 32.6, p<0.001), with 53.1% of Recruited having taken part in 1 or more previous studies vs.32.2% of Refusers. There were no significant differences for any of the other factors.

Analyses between the sub-groups of Non-responders (passive refusal, n= 162) and Responders who then declined (active refusal, n= 152) showed no significant differences in any of the characteristics analysed.

For peer review only - http://bmjopen.bmj.com/site/about/guidelines.xhtml

Table 1: Characteristics of the young people who were approached and comparison between Recruited and Refusers.

Characteristic	Category	Approached n= 410 (%)	Recruited n= 96 (%)	Refusers n= 314 (%)	χ^2 (differences between Recruited and Refusers)
**Sex**	Male	245	53 (55.2%)	192 (61.1%)	χ^2= 1.08 p= 0.22
	Female	165	43 (44.8%)	122 (38.9%)	
**Age when first approached**	Mean (SD)	16.36(1.34)	16.13 (1.22)	16.44 (1.37)	U[a]= 17,032.5 p= 0.05*
**Home location**	Urban	296	67 (69.8%)	229 (73.2%)	χ^2= 0.42 p< 0.51
	Rural	113	29 (30.2%)	84 (26.8%)	
	Missing	1			
**CP subtype**	Spastic unilateral	198 (48.3%)	45 (46.9%)	153 (48.7%)	χ^2= 1.42 p= 0.70
	Spastic bilateral	196 (47.8%)	48 (50.0%)	148 (47.1%)	
	Not spastic (dyskinesia, ataxia, unclassified)	16 (3.9%)	3 (3.1%)	13 (4.2%)	
**GMFCS**	Level I	106 (25.9%)	22 (23.4%)	84 (27.3%)	χ^2= 1.31 p< 0.86
	Level II	191 (46.6%)	44 (46.8%)	147 (47.7%)	
	Level III	65 (15.9%)	18 (19.1%)	47 (15.3%)	
	Level IV	26 (6.3%)	7 (7.4%)	19 (6.2%)	
	Level V	14 (3.4%)	3 (3.2%)	11 (3.6%)	
	Missing	8 (2.0%)			
**Intellectual impairment**	IQ >70	345 (84.1%)	81 (87.1%)	264 (85.4%)	χ^2= 0.16 p< 0.68
	IQ 50-69	57 (13.9%)	12 (12.9%)	45 (14.6%)	
	Missing	8 (2.0%)			
**Approach method**	Direct (NECCPS)	224	49 (51.0%)	175 (55.4%)	χ^2= 0.65 p= 0.41
	Indirect (NICPR)	186	47 (49.0%)	139 (44.3%)	
**Approached about previous studies**	0	153 (37.3%)	31 (32.3%)	122 (38.9%)	χ^2= 10.19 p< 0.11
	1	86 (21.0%)	17 (17.7%)	69 (22.0%)	
	2	112 (27.3%)	31 (32.3%)	81 (25.8%)	
	>3	59 (14.4%)	17 (17.7%)	42 (13.3%)	
**Recruited to previous studies**	0	258 (62.9%)	45 (46.9%)	213 (67.8%)	χ^2= 32.61 p< 0.000*
	1	66 (16.1%)	16 (16.7%)	50 (15.9%)	
	2	56 (13.7%)	21 (21.9%)	35 (11.1%)	
	>3	30 (7.3%)	14 (14.5%)	16 (5.2%)	

*Significant difference; [a]Mann-Whitney U test; NICPR Northern Ireland Cerebral Palsy Register; NECCPS North of England Cerebral Palsy Survey; GMFCS Gross Motor Function Classification System

For peer review only - http://bmjopen.bmj.com/site/about/guidelines.xhtml

Predictors of recruitment to the Transition study

The Hosmer-Lemeshow test found agreement between the observed and the predicted outcomes, indicating the full model was reliable (χ^2=8.1; p= 0.42). The full model was significant in distinguishing between Refusers and Recruited (χ^2=36.8, p=0.004) and accounted for between 8.8% and 13.3% of the variance in refusal, with 96.7% of Refusers successfully predicted. However, the model predicted only 8.6% of those Recruited. Therefore, overall 76.2% of predictions were accurate. Table 2 shows odds rations with 95% CIs for each predictor variable. Only age and the number of previous studies young people had been approached about or taken part in significantly predicted refusal. The ORs values indicated that an increase of 1 year in age at contact was associated with a decrease in the odds of refusal by 0.79. ORs values also indicated that being approached about many studies was associated with a decrease in the odds of refusal, especially if at least three studies (OR 0.11.) In contrast, having been actually recruited to previous studies was associated with an increase in the odds of refusal, odds which rose with the number of studies recruited to.

For peer review only

For peer review only - http://bmjopen.bmj.com/site/about/guidelines.xhtml

Table 2: Factors associated with refusal to take part in the Transition study: logistic regression

Variable	Category	Odds ratio (95% CI)
**Method of approach**	Indirect (NICPR)†	1.00
	Direct (NECCPS)	1.08 (0.5-2.32)
**Sex**	Female†	1.00
	Male	0.67 (0.4-1.12)
**Age when first contacted**		0.79 (0.63-0.98)*
**Home location**	Rural†	1.00
	Urban	0.83 (0.47-1.47)
**CP subtype**	Non-spastic†	1.00
	Spastic unilateral	1.6 (0.38-6.76)
	Spastic bilateral	1.55 (0.38-6.25)
**GMFCS**	Level V†	1.00
	Level I	0.67 (0.14-3.09)
	Level II	0.87 (0.19-3.8)
	Level III	1.09 (0.24-4.82)
	Level IV	0.96 (0.17-5.24)
**Intellectual impairment**	None†	1.00
	Yes	1.22 (0.56-2.67)
**Approached about previous studies**	No studies†	1.00
	One study	0.66 (0.29-1.5)
	Two studies	0.35 (0.11-1.14)
	Three or more studies	0.11 (0.02-0.69)*
**Recruited to previous studies**	No studies†	1.00
	One study	2.95 (1.1-7.91)*
	Two studies	7.45 (2.38-23.33)*
	Three or more studies	34.67 (6.345-189.45)*

*Significant predictor variables in the model $p < 0.05$; †Reference category; NICPR Northern Ireland Cerebral Palsy Register; NECCPS North of England Cerebral Palsy Survey; GMFCS Gross Motor Function Classification System

For peer review only - http://bmjopen.bmj.com/site/about/guidelines.xhtml

DISCUSSION

Of the 431 Eligible young people, 96 (23.4%) were recruited to the Transition study. The proportion of Refusers was 76.6% (n= 314) of whom 39.5% (n= 162) did not respond and 37.1% (n= 152) declined to take part. The majority who declined stated they were not interested in the study (n= 83). Some young people had complex family or health situations and chose not to take part (n= 6) and three young people declined because they had little or no contact with health care services.

Previous studies assessing recruitment bias and predictors for drop-out rate in surveys with children and young people with CP reported higher recruitment rates of between 72.6% and 47.3%.[14, 19-21] Although the refusal rate in those studies was lower, our non-response rate was similar to that reported by Dickinson et al. (36.7%) [18] and marginally higher than that reported by Parkes et al. (31.5%).[19]

The Transition Research Programme involved young people as co-researchers [17] who assisted with aspects of recruitment and retention of participants in the Transition study, e.g. the design of information sheets, consent and refusal forms and ensuring different routes of communication. Despite these efforts, more than half of those Responders who declined reported lack of interest and more than one third of those Approached did not respond. Although adult researchers and young people involved in the public and patient involvement (PPI) work for The Transition Research Programme described positive experiences [17], the impact of their work on recruitment to the Transition study could not be assessed.

Transition from child to adult health care services has been described as a challenging time for young people with complex health care need resulting in issues with non-adherence to health care. [22, 23] This may not be different with research and some young people will not want to engage despite efforts to involve users in the recruitment process. There is evidence on the positive impact and challenges of PPI in health and social care research and its potential to increase recruitment of participants. [24, 25] However, there is a wide variation on how evidence was assessed and reported, and most studies only collected qualitative data [24, 25]. Nevertheless, researchers should consider various recruitment methods and ways in which users can assist with the recruitment of participants. [26, 27] It is also important for researchers to report all stages of recruitment [13] and PPI. [4, 24, 25] Compared to Refusers, a higher percentage of those Recruited to the Transition study had taken part in one or more previous studies (53% versus 32%) and were younger on average. The only factors predicting refusal were age, the number of previous studies eligible participants had been approached about, and the number of studies they had taken part in. Older individuals were less likely to refuse and this may be because they were more independent and making their own

For peer review only - http://bmjopen.bmj.com/site/about/guidelines.xhtml

decisions. Also, the topic of the study may have been of more interest to those who were older. The information sharing and consent process used by researchers would have conveyed to the young people that their opinion was valued, thus increasing their sense of control. [6, 28] However, these findings should be considered with caution as data for age was non normally distributed, with a positive skewness towards younger age in the Recruited group and negative skewness towards older age in the Refusers group; this could explain the small difference in the mean age (0.3 years) between Recruited and Refusers which was only just significant (p= 0.05). In other studies [14, 19-21] investigating recruitment of children and young people with CP, age was not a factor which affected recruitment, but inclusion criteria covered a wide age range (4-25 years) and included participants with all levels of intellectual ability. Indeed, authors reported that those with more complex or severe CP, such as the presence of intellectual problems or not being ambulant, were more likely to take part in their research.[21] This may be because their parents gave consent whereas in the Transition study the focus was on young people giving consent and young people with severe learning disabilities were excluded. This may have contributed to why the recruitment rate was lower than in previous studies. In addition, social networking and fitting in among peers has been shown to be important for adolescents; this may be relevant for those who may have refused to take part in a study simply because it reminds them of their condition.[6]

Research fatigue could also be an issue affecting recruitment. This study is the only one to our knowledge including information on the number of previous studies eligible participants had been approached about or taken part in. Our results showed that young people who had been previously approached about research were less likely to refuse to join our study, and this became even less likely as the number of studies they had been approached about rose. However, those actually recruited to previous research were more likely to refuse to join our study, with more studies making refusal even more likely. Approaching young people about studies can be positive as they may become more aware about research and thus more likely to want to participate in it. However, researchers should be aware of the potential for research fatigue among those recruited to previous studies. Registers such as the NECCPS and the NICPR would benefit from keeping records of studies they have been involved in, who has been contacted about research and their response.

There were no significant differences between Recruited and Refusers regarding the method of approach (Direct vs. Indirect) and the logistic regression model did not find method of approach to be a factor predicting refusal. Therefore, it seems unlikely that different approach methods to contact eligible participants had influenced recruitment to our study. In contrast, previous research has shown that when parents and adolescents have discordant views, adolescents are more likely to

For peer review only - http://bmjopen.bmj.com/site/about/guidelines.xhtml

agree to participate when approached by a clinician they know.[22] Equally, Dickinson et al. [20] found that registers which used the Direct approach had half the refusal rate when compared to those using Indirect methods. However, both NICPR and NECCPS are based in the UK whereas Dickinson's study involved registers from other countries (France, Italy, Sweden, Republic of Ireland and Denmark) with different data protection requirements.

The high non-response rate in this study (those who did not respond at all to the approach) is, similar to rates reported in other studies.[14, 19-21] Thus, there may be a subset of young people with CP who are not interested in research. Further, there were no significant differences in any of their impairments or socio-demographics characteristics between those who declined (active refusal) and Non-responders (passive refusal). Our model only predicted between 9% and 13.7% of the variance in recruitment, and therefore other health and socio-economic factors may influence recruitment. Non-response has been linked to poorer health behaviour and differences in socio-economic background but few studies have focused on adolescents.[4] Mattila et al. [5, 29] found that non-responders to surveys among adolescents in Finland were more likely to engage in negative health behaviours, to suffer mental health disorders and to be more likely to die; these increased risks persisted to age 25 years. Although these studies focused on the general adolescent population, this developmental stage is also a challenging time in the lives of young people with chronic conditions regardless of their diagnoses.[30, 31] It is a time of particular risk for poorer medical outcomes and non-adherence to treatment.[32, 33] As previously discussed, young people may choose not to be involved in research. PPI has the potential to optimised recruitment, although evidence for this is limited. [24, 25]

Strengths and limitations

Both population-based registers used the same definition of CP, classification of CP subtypes and severity, and both confirmed information for cases at age 5 years.[13, 14] Whilst this information was used initially to determine those who were Potentially eligible, subsequent contact brought the information up to date and in particular we found that level of intellectual ability was often no longer accurate.

We could not fully assess how socio-economic status affected recruitment because of the lack of equivalence between the deprivation indices used in Northern Ireland and England. However, it was possible to compare home location (urban vs. rural). Young people who had been approached about or had taken part in three or more studies had to be analysed as a single category due to small numbers. Also, analysis of the effect of previous involvement in research was limited to the number of studies without taking into consideration their nature or the consent procedures. Although the p

For peer review only - http://bmjopen.bmj.com/site/about/guidelines.xhtml

value for those variables showing significant differences was well below p=0.05, there is always a possibility of type I errors.

CONCLUSION

It is encouraging that the two different methods used to approach young people with CP (Direct vs. Indirect) did not affect recruitment. Being older and approached about previous research were associated with increased likelihood of joining the Transition study. However, even in this subset of young people of CP who were research aware and committed to being involved in studies, our findings showed evidence they could experience research fatigue. There is also a subset of young people with CP who are unwilling to take part in research as shown by the high non-response rate reported in this and previous studies. Recruitment of adolescents to research studies remains difficult.

Figure 1 Recruitment flow chart

ACKNOWLEDGEMENTS

#The Transition Collaborative Group consists of: the authors of this paper; other co-applicants: Caroline Bennett, Gail Dovey-Pearce, Ann Le Couteur, Helen McConachie, Janet McDonagh, Jeremy Parr, Mark Pearce, Tim Rapley, Debbie Reape, Luke Vale; advisors: Nichola Chater, Helena Gleeson; local investigators: Anastasia Bem, Stuart Bennett, Amanda Billson, Stephen Bruce, Tim Cheetham, Diana Howlett, Zilla Huma, Maria Lohan, Melanie Meek, Jenny Milne, Julie Owens, Nandu Thalange.

We acknowledge the support of the National Institute for Health Research Clinical Research Network. We are grateful to the young people and their families who participated in the study. We thank the research assistants for their enthusiasm and dedication to contacting young people and collecting high-quality data: Kamar Ameen-Ali, Charlotte George, Kate Hardenberg, Holly Roper, Tracy Scott, Louise Ting, Rose Watson, Hazel Windmill; and we thank Richard Hardy, Alison Mulvenna and Sarah Nolan for software support and administrative support.

FUNDING

This paper summarises independent research funded by the National Institute for Health Research (NIHR) under its Programme Grants for Applied Research scheme (RP-PG-0610-10112). The views expressed are those of the authors and not necessarily those of the UK National Health Service, the NIHR or the Department of Health.

For peer review only - http://bmjopen.bmj.com/site/about/guidelines.xhtml

CONFLICT OF INTEREST

Potential Conflicts of Interest: The authors have no conflicts of interest relevant to this article to disclose

AUTHOR STATEMENT

Authors' contributions: Elena Guiomar García Jalón, Hannah Merrick, Allan Colver, Mark Linden participated in the planning of the study. Elena Guiomar García Jalón had responsibility for data collection with the collaboration of Hannah Merrick. Elena Guiomar García Jalón was responsible for planning and conducting data analysis with the collaboration of Hannah Merrick and Allan Colver. Elena Guiomar García Jalón wrote the drafts of the manuscript with input by Hannah Merrick, Allan Colver, Mark Linden. All authors have seen and approved the version to be published.

DATA AVAILABILITY STATEMENT

No additional data available.

REFERENCES

1. Spigarelli M. Adolescent participation in research. *J Adolesc Health* 2008; 43: 1-2.
2. Middelkoop K, Landon M, Mark D, et al. Adolescent and Adult Participation in an HIV vaccine trial preparedness cohort in South Africa. *J Adolesc Health* 2008; 43: 8-14.
3. Hendricks-Fergunson VL, Cherve BO, Burns DS, et al. Recruitment strategies and rates of a multi-site behavioural intervention for adolescents and young adults with cancer. *J Pediatr Health Care* 2012; 27: 434-42.
4. Vangeepuram N, Townsend K, Arniella G, et al. Recruitment in clinical versus community-based sites for a pilot youth diabetes prevention programme, East Harlem, New York, 2011-2012. *Prev Chronic Dis*; 2016 13 (14): 1-8
5. Mattila VM, Parkkari J, Rimpela A. Adolescent survey non-response and later risk of death. A prospective cohort study of 78,609 persons with 11-year follow-up. *BMC Public Health* 2007; 7: 87.
6. McDonagh JE, Bateman B. Nothing about us without us: considerations for research involving young people. *Arch Dis Child (Education and Practice)* 2012; 97: 55-60.
7. van Staa A, Jedeloo S, Latour JM, et al. Exciting but exhausting: experiences with participatory research with chronically ill adolescents. *Health Expec* 2010; 13(1):95-107.

For peer review only

For peer review only - http://bmjopen.bmj.com/site/about/guidelines.xhtml

8. McCullagh S, Feinstein A. Outcome after mild traumatic brain injury: an examination of recruitment bias. *J NeurolNeurosur Psych* 2003; 74: 39-43.

9. Rahi JS, Manara I, Tuomainen H, et al. Engaging families in health services research on childhood visual impairment: barriers to, and degree and nature of bias in, participation. *Brit J Ophthalmol* 2004; 88: 782-7.

10. Al-Shahi R, Vousden C, Warlow C, for the Scottish Intracranial Vascular Malformation Study (SIVMS) Steering Committee. Bias from requiring explicit consent from all participants in observational research: prospective, population based study. *BMJ* 2005; 31: 942-5.

11. Hille ETM, Elbertse L, Gravenhorst JB, et al. Non-response bias in a follow-up study of 19-year-old adolescents born as preterm infants. *Pediatrics* 2005; 116: 622-66.

12. Junghans C, Feder G, Hemingway H, et al. Recruiting patients to medical research: double blind randomized trial of ‘opt-in’ versus ‘opt-out’ strategies. *BMJ* 2005; 33: 940-2.

13. Hudson FB, Oostendorp LJM, Candy B, et al. The under reporting of recruitment strategies in research with children with life-threatening illnesses: A systematic review. *Palliat Med* 2017; 31 (5): 419-36.

14. Parkes J, Kerr C, McDowell BC, et al. Recruitment bias in a population-based study of children with cerebral palsy. *Pediatrics* 2006; 118: 1616-22.

15. Colver AF, Gibson M, Hey EN, et al. Increasing rates of cerebral palsy across the severity spectrum in north-east England 1964-1993. The North of England Collaborative Cerebral Palsy Survey. *Arch Dis Child*. *Fetal Neonatal Edition* 2000; 83: F7–12.

16. Parkes J, Dolk H, Hill N, et al. Cerebral palsy in Northern Ireland: 1981-93. *Paediatr Perinat Ep* 2001; 15: 278-86.

17. Dovey-Pearce G, Walker S, Fairgrieve S, et al. The burden of proof: The process of involving young people in research. *Health Expect* 2019; 00: 1-10.

18. Palisano R, Rosenbaum P, Walter S, et al. Development and reliability of a system to classify gross motor function in children with cerebral palsy. *Dev Med Child Neurol* 1997; 39: 214–23.

19. Parkes J, Donnelly C, McDowell B, et al. Recruitment bias and characteristics of participants with severe cerebral palsy in a cross-sectional survey. *J AdvNurs* 2012; 68: 368–78.

For peer review only - http://bmjopen.bmj.com/site/about/guidelines.xhtml

20. Dickinson H, Parkinson K, McManus V, et al. Assessment of data quality in a multi-centre cross-sectional study of participation and quality of life of children with cerebral palsy. *BMC Public Health* 2006; 6: 273.

21. Dickinson HO, Rapp M, Arnaud C, et al. Predictors of drop-out in a multi-centre longitudinal study of participation and quality of life of children with cerebral palsy. *BMC Research Notes* 2012*;* 15: 300.

22. McDonagh JE and Kelly DA. The challenges and opportunities for transitional care research. *Pediatric Transplant* 2010*;* 14: 688-700

23. Watson R Parr JR, Joyce C, et al. Models of transitional care for young people with complex health needs: a scoping review. *Child Care Health Dev*, 2011; 37 (6): 780-91

24. Exploring the impact of public involvement on the quality of research: examples. INVOLVE Web site. http://www.invo.org.uk/the-quality-of-research/. Accessed 18th February, 2020.

25. Brett J, Staniszewska S, Mockford C, et al. Mapping the impact of patient and public involvement on health and social care research: a systematic review. *Health Expectations* 2012; 17: 637–50

26. Preston J and Moneypenny S. Involving young people in the design and delivery of health research. INVOLVE Web site http://www.invo.org.uk/involvingyoung-people-in-the-design-and-delivery-of-health-research/ Accessed: 18th February, 2020.

27. Sanders J and Munford R. Hidden in plain view: finding and enhancing the population of marginalized young people in research. *Int J Qual Meth* 2017; 16 (1): 1-12.

28. Brody JL, Annett RD, Scherer DG, et al. Enrolling adolescents in asthma research: adolescent, parent and physician influence in the decision-making process. *J Asthma* 2009; 49: 492-7.

29. Mattila V, Parkkari J, Kannus P, et al. Occurrence and risk factors of unintentional injuries among 12 to 18 year old Finns – a survey of 8219 adolescents. *Eur J Epidemiol* 2004; 19: 437-44.

30. Bolton-Maggs PH. Transition of care from paediatric to adult services in haematology. *Arch Dis Child* 2007; 92: 797-801.

31. JordanA, McDonagh JE. Recognition of emerging adulthood in UK rheumatology: the case for young adult rheumatology service developments. *Rheumatology (Oxford)* 2007; 46: 188-91.

For peer review only

For peer review only - http://bmjopen.bmj.com/site/about/guidelines.xhtml

32. Reed-Knight B, Blount RL, Gilleland J. The transition of health care responsibility from parents to youth diagnosed with chronic illness: a developmental systems perspective. *Fam Syst Health* 2014; 32: 219-34.

33. Fredericks EM, Magee JC, Eder SJ, et al. Quality improvement targeting adherence during the transition from a pediatric to adult liver transplant clinic. *J ClinPsychol Med Settings* 2015; 22: 150-9.

For peer review only

For peer review only - http://bmjopen.bmj.com/site/about/guidelines.xhtml

Figure 1 Recruitment flow chart

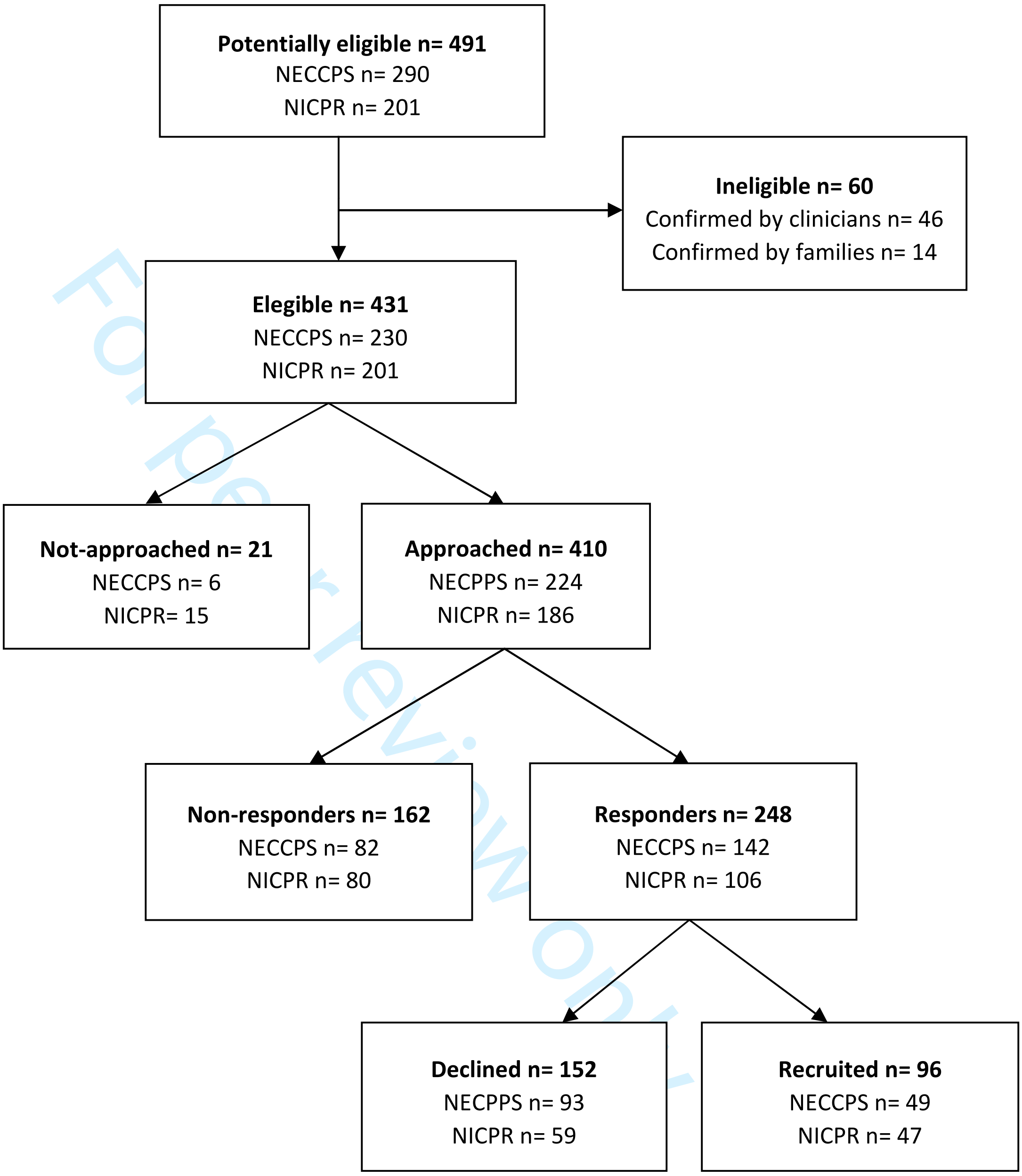

NICPR Northern Ireland Cerebral Palsy Register – Indirect approach
NECCPS North of England Cerebral Palsy Survey – Direct approach

STROBE Statement—Checklist of items that should be included in reports of ***cross-sectional studies***

	Item No	Recommendation	Page No
**Title and abstract**	1	(*a*) Indicate the study's design with a commonly used term in the title or the abstract	1
		(*b*) Provide in the abstract an informative and balanced summary of what was done and what was found	2
**Introduction**			
Background/rationale	2	Explain the scientific background and rationale for the investigation being reported	3
Objectives	3	State specific objectives, including any prespecified hypotheses	3
**Methods**			
Study design	4	Present key elements of study design early in the paper	3
Setting	5	Describe the setting, locations, and relevant dates, including periods of recruitment, exposure, follow-up, and data collection	3-4
Participants	6	(*a*) Give the eligibility criteria, and the sources and methods of selection of participants	3-4
Variables	7	Clearly define all outcomes, exposures, predictors, potential confounders, and effect modifiers. Give diagnostic criteria, if applicable	4
Data sources/ measurement	8*	For each variable of interest, give sources of data and details of methods of assessment (measurement). Describe comparability of assessment methods if there is more than one group	4
Bias	9	Describe any efforts to address potential sources of bias	-
Study size	10	Explain how the study size was arrived at	5
Quantitative variables	11	Explain how quantitative variables were handled in the analyses. If applicable, describe which groupings were chosen and why	4
Statistical methods	12	(*a*) Describe all statistical methods, including those used to control for confounding	5
		(*b*) Describe any methods used to examine subgroups and interactions	5
		(*c*) Explain how missing data were addressed	-
		(*d*) If applicable, describe analytical methods taking account of sampling strategy	NA
		(*e*) Describe any sensitivity analyses	-
**Results**			
Participants	13*	(a) Report numbers of individuals at each stage of study—eg numbers potentially eligible, examined for eligibility, confirmed eligible, included in the study, completing follow-up, and analysed	5
		(b) Give reasons for non-participation at each stage	5
		(c) Consider use of a flow diagram	Separate file uploaded
Descriptive data	14*	(a) Give characteristics of study participants (eg demographic, clinical, social) and information on exposures and potential confounders	6
		(b) Indicate number of participants with missing data for each variable of interest	6
Outcome data	15*	Report numbers of outcome events or summary measures	6

For peer review only - http://bmjopen.bmj.com/site/about/guidelines.xhtml

Main results	16	(*a*) Give unadjusted estimates and, if applicable, confounder-adjusted estimates and their precision (eg, 95% confidence interval). Make clear which confounders were adjusted for and why they were included	5, 7, 8
		(*b*) Report category boundaries when continuous variables were categorized	
		(*c*) If relevant, consider translating estimates of relative risk into absolute risk for a meaningful time period	
Other analyses	17	Report other analyses done—eg analyses of subgroups and interactions, and sensitivity analyses	7
**Discussion**			
Key results	18	Summarise key results with reference to study objectives	9-10
Limitations	19	Discuss limitations of the study, taking into account sources of potential bias or imprecision. Discuss both direction and magnitude of any potential bias	11
Interpretation	20	Give a cautious overall interpretation of results considering objectives, limitations, multiplicity of analyses, results from similar studies, and other relevant evidence	11
Generalisability	21	Discuss the generalisability (external validity) of the study results	-
**Other information**			
Funding	22	Give the source of funding and the role of the funders for the present study and, if applicable, for the original study on which the present article is based	12

*Give information separately for exposed and unexposed groups.

Note: An Explanation and Elaboration article discusses each checklist item and gives methodological background and published examples of transparent reporting. The STROBE checklist is best used in conjunction with this article (freely available on the Web sites of PLoS Medicine at http://www.plosmedicine.org/, Annals of Internal Medicine at http://www.annals.org/, and Epidemiology at http://www.epidem.com/). Information on the STROBE Initiative is available at www.strobe-statement.org.

For peer review only
